# *In silico* profiling of *Escherichia coli* and *Saccharomyces cerevisiae* as terpenoid factories

**DOI:** 10.1186/1475-2859-12-84

**Published:** 2013-09-23

**Authors:** Evamaria Gruchattka, Oliver Hädicke, Steffen Klamt, Verena Schütz, Oliver Kayser

**Affiliations:** 1 Technical Biochemistry, Department of Biochemical and Chemical Engineering, TU Dortmund University, Emil-Figge-Str. 66, 44227 Dortmund, Germany; 2 Analysis and Redesign of Biological Networks, Max Planck Institute for Dynamics of Complex Technical Systems, Sandtorstr. 1, 39106 Magdeburg, Germany; 3 Biomax Informatics AG, Robert-Koch-Str. 2, 82152 Planegg, Germany

**Keywords:** Terpenoids, Isoprenoids, *In silico*, Elementary mode analysis, Constrained minimal cut sets, Metabolic engineering, *Escherichia coli*, *Saccharomyces cerevisiae*

## Abstract

**Background:**

Heterologous microbial production of rare plant terpenoids of medicinal or industrial interest is attracting more and more attention but terpenoid yields are still low. *Escherichia coli* and *Saccharomyces cerevisiae* are the most widely used heterologous hosts; a direct comparison of both hosts based on experimental data is difficult though. Hence, the terpenoid pathways of *E. coli* (via 1-deoxy-D-xylulose 5-phosphate, DXP) and *S. cerevisiae* (via mevalonate, MVA), the impact of the respective hosts metabolism as well as the impact of different carbon sources were compared *in silico* by means of elementary mode analysis. The focus was set on the yield of isopentenyl diphosphate (IPP), the general terpenoid precursor, to identify new metabolic engineering strategies for an enhanced terpenoid yield.

**Results:**

Starting from the respective precursor metabolites of the terpenoid pathways (pyruvate and glyceraldehyde-3-phosphate for the DXP pathway and acetyl-CoA for the MVA pathway) and considering only carbon stoichiometry, the two terpenoid pathways are identical with respect to carbon yield. However, with glucose as substrate, the MVA pathway has a lower potential to supply terpenoids in high yields than the DXP pathway if the formation of the required precursors is taken into account, due to the carbon loss in the formation of acetyl-CoA. This maximum yield is further reduced in both hosts when the required energy and reduction equivalents are considered. Moreover, the choice of carbon source (glucose, xylose, ethanol or glycerol) has an effect on terpenoid yield with non-fermentable carbon sources being more promising. Both hosts have deficiencies in energy and redox equivalents for high yield terpenoid production leading to new overexpression strategies (heterologous enzymes/pathways) for an enhanced terpenoid yield. Finally, several knockout strategies are identified using constrained minimal cut sets enforcing a coupling of growth to a terpenoid yield which is higher than any yield published in scientific literature so far.

**Conclusions:**

This study provides for the first time a comprehensive and detailed *in silico* comparison of the most prominent heterologous hosts *E. coli* and *S. cerevisiae* as terpenoid factories giving an overview on several promising metabolic engineering strategies paving the way for an enhanced terpenoid yield.

## Background

Terpenoids are a class of natural products with important medicinal and industrial applications such as artemisinin (antimalarial) or paclitaxel (anticancer) [1,2]. However, several of these bioactive compounds are scarce and produced only in low amounts in plants, which makes the production in their native hosts uneconomical and environmentally destructive [2]. The total chemical synthesis of many terpenoids is challenging due to their complex structure and neither ecologically nor economically efficient [3-5]. Alternatively, the use of a microbial platform organism for the production of terpenoids may offer the possibility of large‒scale, cost‒effective and environmentally friendly industrial production independent from climate or cultivation risks. Today, *Escherichia coli* and *Saccharomyces cerevisiae* are the most widely used microorganisms for heterologous terpenoid production. They are preferably used due to advanced molecular biology tools, growth speed and their well-established use in industrial biotechnology [5,6]. While most studies for the production of carotenoid compounds of lower complexity such as lycopene (an antioxidative carotenoid) have been carried out in *E. coli*, the trends concerning the production of artemisinin or paclitaxel show a more even distribution between these two hosts with an increasingly more prevalent use of *S. cerevisiae*[5]. This trend might partly be attributed to the fact that *S. cerevisiae* as an eukaryotic organism has the advantage of being more suitable than *E. coli* for the expression of membrane-bound plant cytochrome P450 enzymes along with their respective reductase [2] required for the functionalization of a series of terpenoids [7-9]. A further advantage is the possibility to harness different subcellular compartments [10]. Moreover, yeast can withstand reduced pH and high osmotic pressure [11] and is not susceptible to phage infections.

The production of terpenoids in both organisms requires a sufficient supply of their common precursor isopentenyl diphosphate (IPP) and its isomer dimethylallyl diphosphate (DMAPP), which are then condensed, cyclized etc. leading to the structural diversity of terpenoids. *E. coli* and *S. cerevisiae* differ in their metabolic routes to supply these precursors. The 1-deoxy-D-xylulose 5-phosphate (DXP) pathway of *E. coli* (often also called 2C-methyl-D-erythriol 4-phosphate pathway, MEP) is fed by pyruvate and glyceraldehyde-3-phosphate whereas in the mevalonate (MVA) pathway of *S. cerevisiae* IPP derives from acetyl-CoA [12,13].

Attempts to engineer the DXP pathway in *E. coli* mainly focused on the combinatorial overexpression of the rate limiting enzymes encoded by *dxs*, *idi*, *dxr*, *ispD* and *ispF* with various ranges of expression levels. Those studies stressed the need to carefully balance the expression of the DXP pathway genes with the heterologous terpenoid forming pathway as well as with the host’s overall metabolism [14-21]. Alternatively, various examples show that the introduction of an optimized MVA pathway efficiently increases terpenoid production in *E. coli*, circumventing the largely unidentified regulations of its native DXP pathway and also decoupling the MVA pathway from its native control in yeast [17,22-25].

Numerous engineering strategies have been applied especially to the MVA pathway in *S. cerevisiae*[11]. Three particularly successful interventions have been described in literature: i) overexpression of a truncated version of 3-hydroxy-3-methylglutaryl-coenzyme A reductase (*HMG1*), the key regulatory enzyme of the pathway, devoid of feedback inhibition by farnesyl diphosphate (FPP) [26-28]; ii) downregulation of squalene synthase (*ERG9*) to reduce the flux draining of FPP to biosynthetically related sterols [29,30]; and iii) expression of a mutant transcription factor (*upc2-1*) to transcriptionally up-regulate several MVA pathway genes [7,8,31].

A direct *in vivo* head-to-head comparison of both organisms and terpenoid pathways is difficult to interpret, especially if it is considered that the expression of enzymes of both IPP forming pathways has to be balanced carefully for optimal precursor supply and that *E. coli* and *S. cerevisiae* differ in their central metabolic networks. However, *in silico* methods can reveal their theoretical potential and give further insight into the properties of the underlying metabolic networks, thus providing a rational basis for metabolic engineering.

Elementary mode analysis (EMA) is a key methodology to study essential properties of a metabolic network. Elementary modes (EMs) characterize the space of feasible steady-state flux distributions; any such flux distribution can be represented as a non-negative (conic) linear combination of elementary modes. Based on the stoichiometric matrix, the steady-state assumption, thermodynamic constraints (reaction reversibilities) and a non-decomposability constraint, elementary modes can be calculated without the need for kinetic data, *a priori* measurements, or an objective function [32-36]. EMA has been applied to calculate the overall capacity of a given metabolic network, e.g. the theoretical maximum yield under a given genetic constitution or on different substrates [32,37] in order to estimate the potential efficiency of a biotechnological process. Moreover, it has been successfully used as a basis for the computation of intervention strategies to obtain superior production strains [32,38-41]. Recently, Hädicke *et al*. [40] introduced the concept of constrained minimal cut sets (cMCSs) to compute knockout strategies for coupled product and biomass synthesis allowing not only the specification of functionalities that are to be disabled in the metabolic network but also of those that have to be preserved. Furthermore, all equivalent knockout strategies to accomplish the same engineering goal are computed, giving the researcher the flexibility to choose the best combination of gene deletions in terms of practical realization. cMCSs comprise and extend e.g. the approach of minimal metabolic functionality [42] that has been successfully applied multiple times in order to identify metabolic engineering strategies [41-44].

In this study, EMA is applied to compare the metabolic networks of *E. coli* and *S. cerevisiae* for the production of the common terpenoid precursor IPP by means of carbon stoichiometry, energy and redox equivalent requirements. Further, the potential of the two hosts’ central metabolic networks to meet those requirements if the MVA, the DXP or both pathways are operating is analyzed. Moreover, the impact of different industrially relevant carbon sources on IPP production is investigated *in silico*.

While most studies on terpenoid production mainly focused on engineering the DXP and MVA pathway, only a few studies deal with the identification of engineering targets in the two host’s central metabolism for an improved precursor supply [31,41,45-50] leaving tremendous room for further improvements. Therefore, the impact of heterologously introduced enzymes is analyzed *in silico* and knockout targets are computed based on constrained minimal cut sets to give guidelines for metabolic engineering in order to obtain a highly efficient IPP platform organism.

## Results and discussion

This section is conceptually divided into two parts. The first subsection focuses on the analysis of the two terpenoid pathways starting with a comparison of the DXP and MVA pathway independent from their host organism. Thereafter, the stoichiometric potential of *E. coli* and *S. cerevisiae* to produce the precursor IPP is analyzed. For this purpose, models of the central carbon metabolism were built up and analyzed (65 reactions and 50 metabolites for *E. coli* and 69 reactions and 60 metabolites for *S. cerevisiae*, both for growth on glucose, see Methods section and Additional file 1 for details). Subsequently, the optimal flux distributions are characterized and the impact of an interchange of the pathways of *E. coli* and *S. cerevisiae* is investigated *in silico*. The pathway analysis part is completed by analyzing alternative substrates and comparing the identified theoretically optimal yields with available literature data. The results of all these calculations are summarized in Table 1.

**Table 1:** **Overview of *in silico *computations: number of elementary modes (EMs) and maximal IPP and biomass carbon yields on different carbon sources [C-mol/C-mol] for computations for *E. coli *and *S. cerevisiae***

**Organism**	**Carbon source**	**Characteristics**	**EMs**	**Max IPP yield at zero growth**	**Max IPP yield with growth**	**Max biomass carbon yield**
*E. coli*	Glucose	Wild type	36,590	0.67	0.57	0.85
*E. coli*	Glucose	Wild type, anaerobic	8,343	0.48	0.41	0.35
*E. coli*	Glucose	ATP source	48,603	0.71	0.57	0.99
*E. coli*	Glucose	NADH source	45,570	0.83	0.57	0.99
*E. coli*	Glucose	NADPH source	44,842	0.83	0.57	0.99
*E. coli*	Glucose	GAP-DH (NADP^+^)	74,651	0.71	0.62	0.99
*E. coli*	Glucose	MVA only	35,968	0.56	0.54	0.85
*E. coli*	Glucose	MVA + DXP	44,850	0.67	0.59	0.85
*E. coli*	Glycerol	Wild type	26,160	0.78	0.77	0.99
*E. coli*	Glycerol	ATP source	29,088	0.83	0.77	0.99
*E. coli*	Glycerol	NADH source	29,126	0.83	0.77	0.99
*E. coli*	Glycerol	NADPH source	28,252	0.83	0.77	0.99
*E. coli*	Glycerol	GAP-DH (NADP^+^)	62,665	0.83	0.77	0.99
*E. coli*	Glycerol	MVA only	22,225	0.56	0.45	0.99
*E. coli*	Glycerol	MVA + DXP	28,326	0.78	0.77	0.99
*S. cerevisiae*	Glucose	Wild type	9,844	0.53	0.39	0.80
*S. cerevisiae*	Glucose	Wild type, anaerobic	495	0.10	0.09	0.21
*S. cerevisiae*	Glucose	ATP source	15,326	0.56	0.39	0.89
*S. cerevisiae*	Glucose	NADH source	12,080	0.56	0.39	0.89
*S. cerevisiae*	Glucose	PDH	17,943	0.56	0.51	0.81
*S. cerevisiae*	Glucose	Mitochondrial MVA only	10,232	0.56	0.51	0.80
*S. cerevisiae*	Glucose	Mitochondrial + cytosolic MVA	128,427	0.56	0.53	0.82
*S. cerevisiae*	Glucose	ACL	17,155	0.55	0.39	0.80
*S. cerevisiae*	Glucose	DXP only	8,865	0.64	0.57	0.80
*S. cerevisiae*	Glucose	DXP + MVA	11,738	0.64	0.57	0.80
*S. cerevisiae*	Glucose	DXP + ATP source	13,731	0.66	0.57	0.89
*S. cerevisiae*	Glucose	DXP + NADH source	10,714	0.66	0.57	0.89
*S. cerevisiae*	Glucose	DXP + NADPH source	16,754	0.80	0.57	0.89
*S. cerevisiae*	Glucose	DXP + TH	25,254	0.67	0.57	0.81
*S. cerevisiae*	Glucose	DXP + GAP-DH (NADP^+^)	28,383	0.67	0.57	0.81
*S. cerevisiae*	Galactose	Wild type	9,844	0.53	0.39	0.80
*S. cerevisiae*	Fructose	Wild type	9,844	0.53	0.39	0.80
*S. cerevisiae*	Xylose	XI	6,330	0.53	0.39	0.80
*S. cerevisiae*	Xylose	XR-XDH	16,911	0.53	0.51	0.80
*S. cerevisiae*	Xylose	XI + DXP only	7,364	0.64	0.42	0.80
*S. cerevisiae*	Xylose	XI + DXP + MVA	9,088	0.64	0.44	0.80
*S. cerevisiae*	Xylose	XR-XDH + DXP only	16,513	0.64	0.42	0.80
*S. cerevisiae*	Xylose	XR-XDH + DXP + MVA	20,337	0.64	0.51	0.80
*S. cerevisiae*	Glycerol	Wild type	2,648,133	0.56	0.47	0.87
*S. cerevisiae*	Glycerol	Wild type w/o glyoxylate cycle	5,377	0.56	0.35	0.87
*S. cerevisiae*	Glycerol	DXP only	2,401,411	0.67	0.60	0.87
*S. cerevisiae*	Glycerol	DXP + MVA	4,038,007	0.67	0.60	0.87
*S. cerevisiae*	Glycerol	DXP + NADPH source	3,735,984	0.83	0.80	0.94
*S. cerevisiae*	Glycerol	DXP + GAP-DH (NADP^+^)	6,153,971	0.76	0.63	0.90
*S. cerevisiae*	Ethanol	Wild type	2,874,235	0.68	0.59	0.78
*S. cerevisiae*	Ethanol	ATP source	3,559,725	0.83	0.59	0.81
*S. cerevisiae*	Ethanol	NADH source	3,635,994	0.83	0.62	0.81
*S. cerevisiae*	Ethanol	DXP only	2,846,217	0.63	0.57	0.78
*S. cerevisiae*	Ethanol	DXP + MVA	3,133,493	0.68	0.59	0.78

GAP-DH (NADP^+^) = NADP^+^-dependent glyceraldehyde-3-phosphate dehydrogenase; PDH = cytosolic pyruvate dehydrogenase complex; ACL = ATP-citrate-lyase; TH = soluble and energy-independent transhydrogenase; XI = xylose isomerase pathway; XR-XDH = xylose reductase and xylitol dehydrogenase pathway.

The second part of the results section is related to target identification for metabolic engineering. Suitable heterologous overexpression candidates are identified and promising knockout strategies are computed by means of cMCSs.

### Terpenoid pathway and metabolic network analysis

#### Stoichiometry of terpenoid pathways

The stoichiometry of both pathways is initially analyzed independently from the respective host organism. The precursors of the DXP pathway are glyceraldehyde-3-phosphate (GAP) and pyruvate (PYR) and the overall stoichiometry of this pathway is given by equation 1.

(1) $$\begin{array}{l} \text{GAP}+\text{PYR}+3\;\text{NADPH}+2\;\text{ATP}\\ \;=\text{IPP}+{\text{CO}}_{2}+3\;{\text{NADP}}^{+}+2\;\text{ADP} \end{array}$$

The MVA pathway is fed by the carbon precursor acetyl-CoA (AcCoA) and its stoichiometry is given by equation 2.

(2) $$\begin{array}{ll} 3 & \text{AcCoA}+2\;\text{NADPH}+3\;\text{ATP}\\ & =\text{IPP}+{\text{CO}}_{2}+2\;{\text{NADP}}^{+}+3\;\text{ADP} \end{array}$$

Both terpenoid pathways lose one mol CO_2_ per mol IPP (C5); therefore they are identical with respect to carbon yield (5/6 = 0.83) when starting from their precursors GAP (C3) and PYR (C3) or AcCoA (C2). Regarding the demand of ATP and NADPH, they show only minor differences as the DXP pathway needs one mol more NADPH but one mol less ATP than the MVA pathway. Hence, *a priori*, one could assume that no significant differences in achievable IPP yields occur between *E. coli* and *S. cerevisiae*.

#### Metabolic potential of wild type E. coli and S. cerevisiae to supply IPP

**Figure 1:**
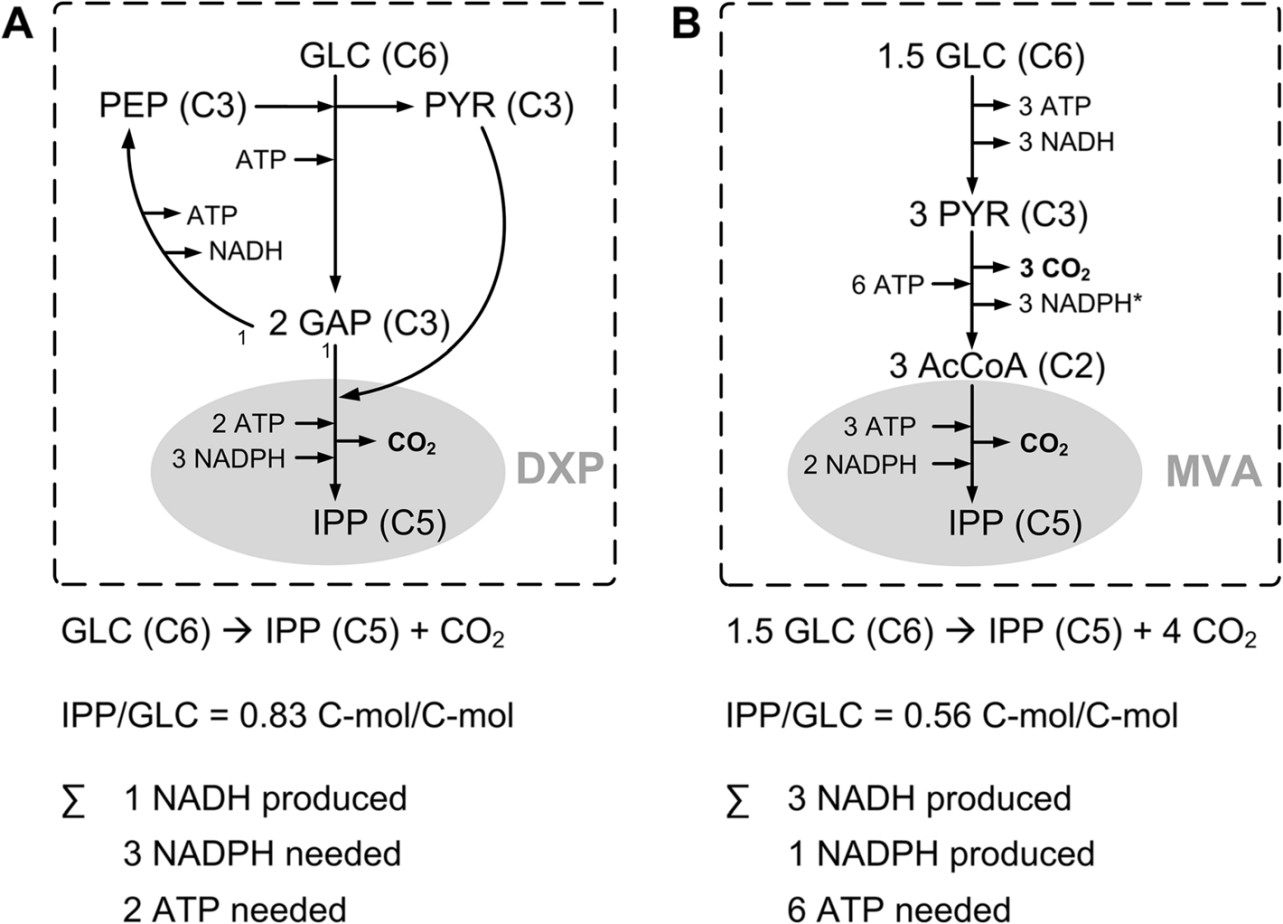
**Comparison of the DXP and MVA pathway. A** section of the central carbon metabolism from glucose (GLC) to IPP: A: of *E. coli* including glycolysis and DXP pathway, and **B**: of *S. cerevisiae* including glycolysis and the MVA pathway. The maximal IPP yields based on carbon stoichiometry and ignoring energy and redox equivalent requirements (5/6 = 0.83 and 5/9 = 0.56 C-mol/C-mol) differ due to different carbon loss via CO_2_ in the formation of the precursors pyruvate (PYR) and glyceraldehyde-3-phosphate (GAP) or acetyl-CoA (AcCoA). The sum shows energy requirements and redox equivalents that have to be balanced by the organism’s metabolism. *: Note that cytosolic NADP^+^-dependent aldehyde dehydrogenase (*ALD6*) is constitutive while cytosolic NAD^+^-dependent aldehyde dehydrogenases (*ALD2*, *ALD3*) are stress-induced, glucose-repressed [51] and were not considered.

Both terpenoid pathways are now analyzed with respect to the metabolic background of their respective host. A first comparison of the maximal IPP yields purely based on carbon stoichiometry, ignoring energy and redox equivalent requirements (i.e. when ATP and NAD(P)H are initially considered to be external metabolites in the model), shows a significant difference. With energy and redox equivalents in excess, the maximum IPP yield on glucose via the DXP pathway in *E. coli* is 5/6 = 0.83 C-mol/C-mol compared to 5/9 = 0.56 C-mol/C-mol in *S. cerevisiae* if the MVA pathway is used (Figure 1). This is due to different amounts of carbon loss via CO_2_ in the formation of the terpenoid pathway precursors. The formation of one mol acetyl-CoA, the precursor of the MVA pathway, includes the generation (and thus carbon loss) of one mol CO_2_. In contrast, the formation of one mol pyruvate and one mol glyceraldehyde-3-phosphate, the precursors of the DXP pathway, does not involve a carbon loss via CO_2_.

Besides the different carbon use efficiencies, the energy and redox equivalent requirements for IPP synthesis differ in *E. coli* and *S. cerevisiae* as well (Figure 1)*.* For the formation of one mol IPP from glucose (GLC) in *E. coli*, the overall stoichiometry is given by equation 3.

(3) $$\begin{array}{l} \text{GLC}+2\;\text{ATP}+3\;\text{NADPH}+{\text{NAD}}^{+}\\ \;=\text{IPP}+{\text{CO}}_{2}+2\;\text{ADP}+3\;{\text{NADP}}^{+}+\text{NADH} \end{array}$$

In *S. cerevisiae*, the overall stoichiometry for synthesizing one mol IPP with glucose as substrate via the MVA pathway (considering only the constitutive NADP^+^-dependent aldehyde dehydrogenase, *ALD6*) is given by equation 4.

(4) $$\begin{array}{ll} \;1.5 & \text{GLC}+3\;\text{ATP}+{\text{NADP}}^{+}+3\;{\text{NAD}}^{+}\\ & =\text{IPP}+4\;{\text{CO}}_{2}+3\;\text{AMP}+\text{NADPH}+3\;\text{NADH} \end{array}$$

Now, in the context of the metabolic background, significant differences are observed. When the pathways are considered isolated from the network, they can both serve as redox sinks as they require reduced redox equivalents (see equations 1 and 2). However, due to the synthesis of the specific pathway precursors, different energy and redox equivalent requirements have to be balanced by the organism’s metabolism. In *E. coli*, the synthesis of IPP is still accompanied by oxidation of NADPH (equation 3) whereby in *S. cerevisiae* reduced redox equivalents are generated (equation 4). In both organisms, additional ATP has to be generated from the substrate for IPP synthesis, which leads to a further reduction of the maximum IPP yield.

**Figure 2:**
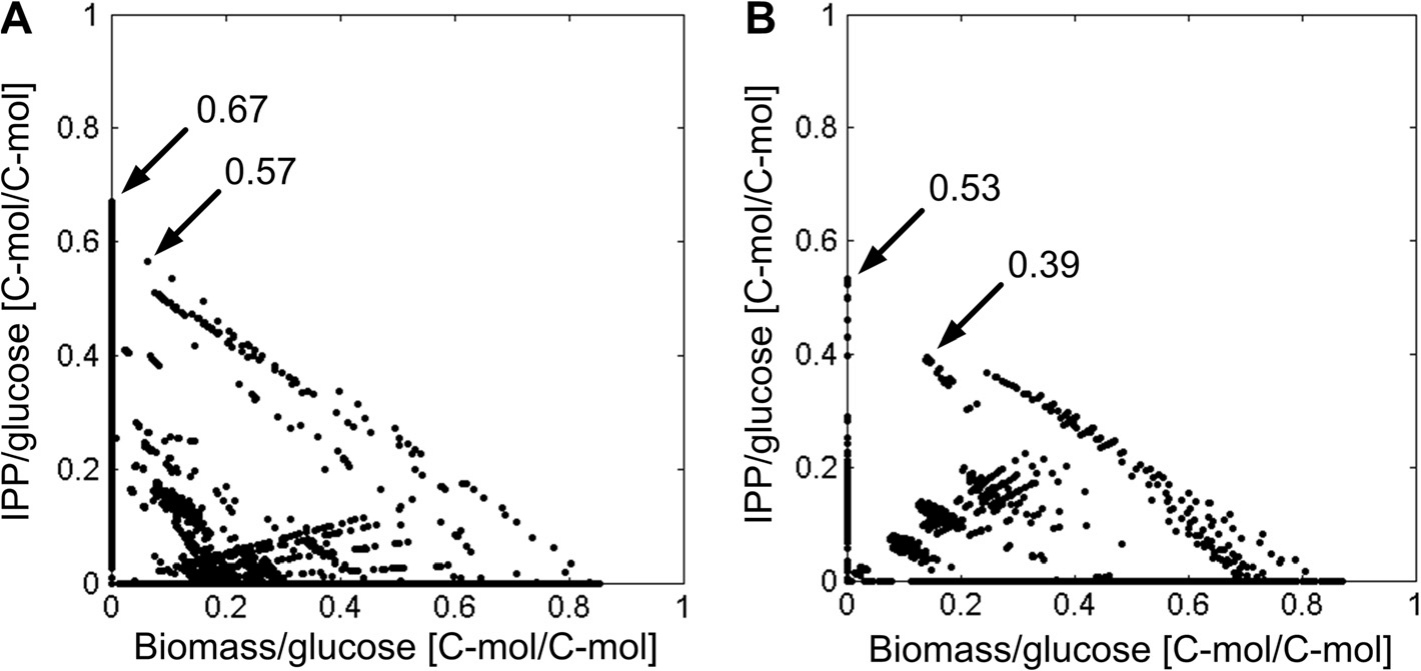
**Comparison of the solution space spanned by elementary modes. A**: Wild type network of *E. coli* (36,590 EMs). **B**: Wild type network of *S. cerevisiae* (9,844 EMs). The biomass yield on glucose is plotted against the IPP yield on glucose [C-mol/C-mol] for all computed elementary modes. Elementary modes on the axes represent modes producing either only biomass (x-axis) or only IPP (y-axis). Theoretical maximal IPP yields at zero growth as well as with biomass formation are highlighted.

Taking the cofactors explicitly into account requires a network-wide view of all pathways. Therefore, elementary mode analysis was performed on the metabolic networks of *E. coli* (DXP pathway) and *S. cerevisiae* (MVA pathway) wild types with glucose as the single carbon source. In total, 36,590 elementary modes were obtained for the *E. coli* network and 9,844 for the *S. cerevisiae* network (Figure 2). Of these, 2,597 modes (26.4%) of *S. cerevisiae* and even 7,675 modes (21.0%) of *E. coli* include IPP formation. Computed elementary modes exhibit different yields for IPP and biomass in both organisms (Figure 2). The maximum IPP yield for *E. coli* is 0.67 C-mol/C-mol and 0.53 C-mol/C-mol for *S. cerevisiae*. If only elementary modes that couple IPP synthesis with biomass formation are considered, the maximum IPP yield drops to 0.57 C-mol/C-mol in *E. coli* and 0.39 C-mol/C-mol in *S. cerevisiae*, respectively.

Hence, *E. coli* has a higher theoretical potential to provide IPP, the common terpenoid precursor.

#### Comparison of flux distributions with maximum IPP production

**Figure 3:**
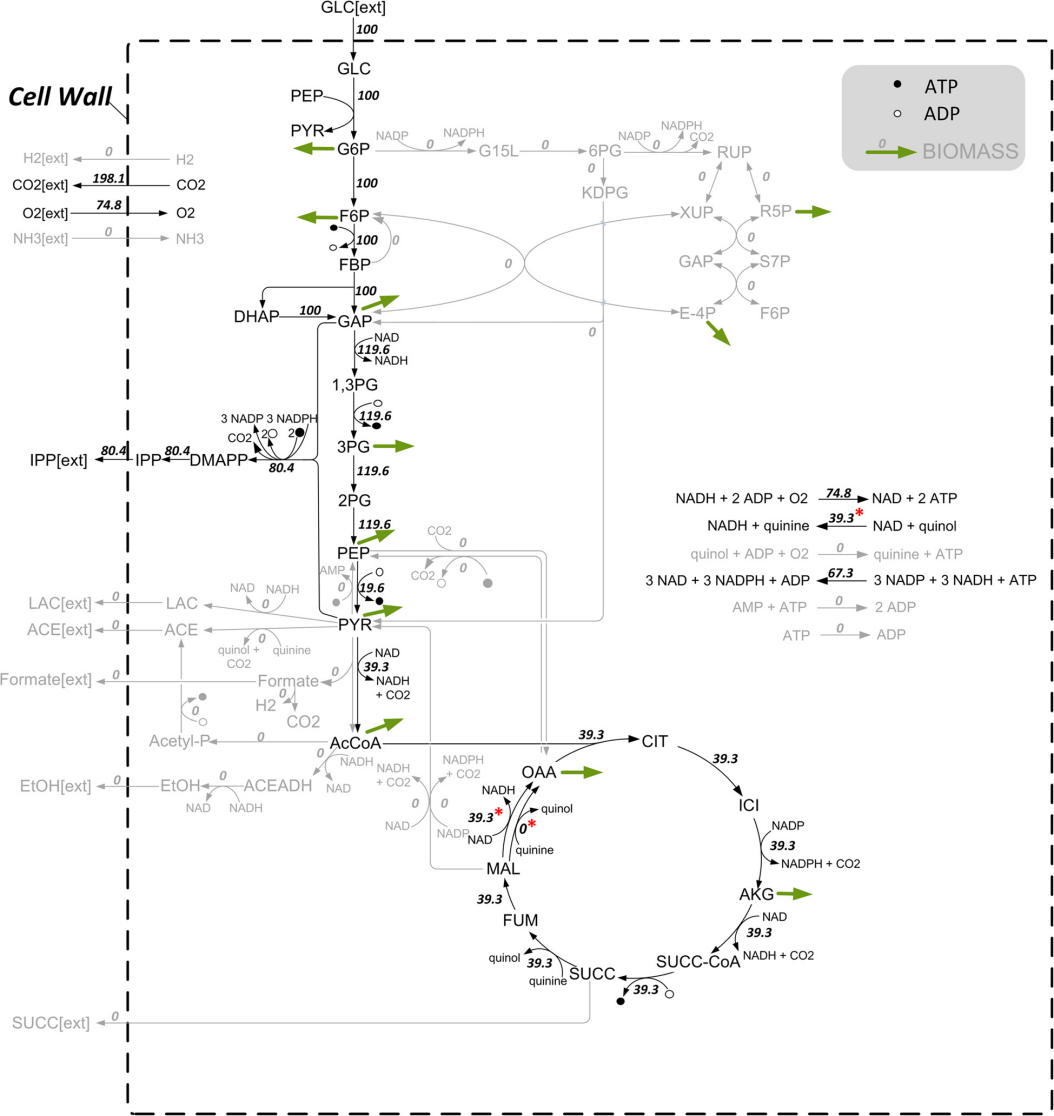
**Optimal flux distribution of *E. coli. ***Wild type (DXP pathway) on glucose as single carbon source without biomass formation (one of two modes with maximum theoretical IPP carbon yield). Numbers indicate relative molar fluxes (mmol/(gCDW h), CDW = cell dry weight) normalized to glucose uptake. Grey reactions are not active (flux = 0). Variable fluxes within two optimal modes are marked with an asterisk (*). Bold green arrows indicate biomass precursors.

**Figure 4:**
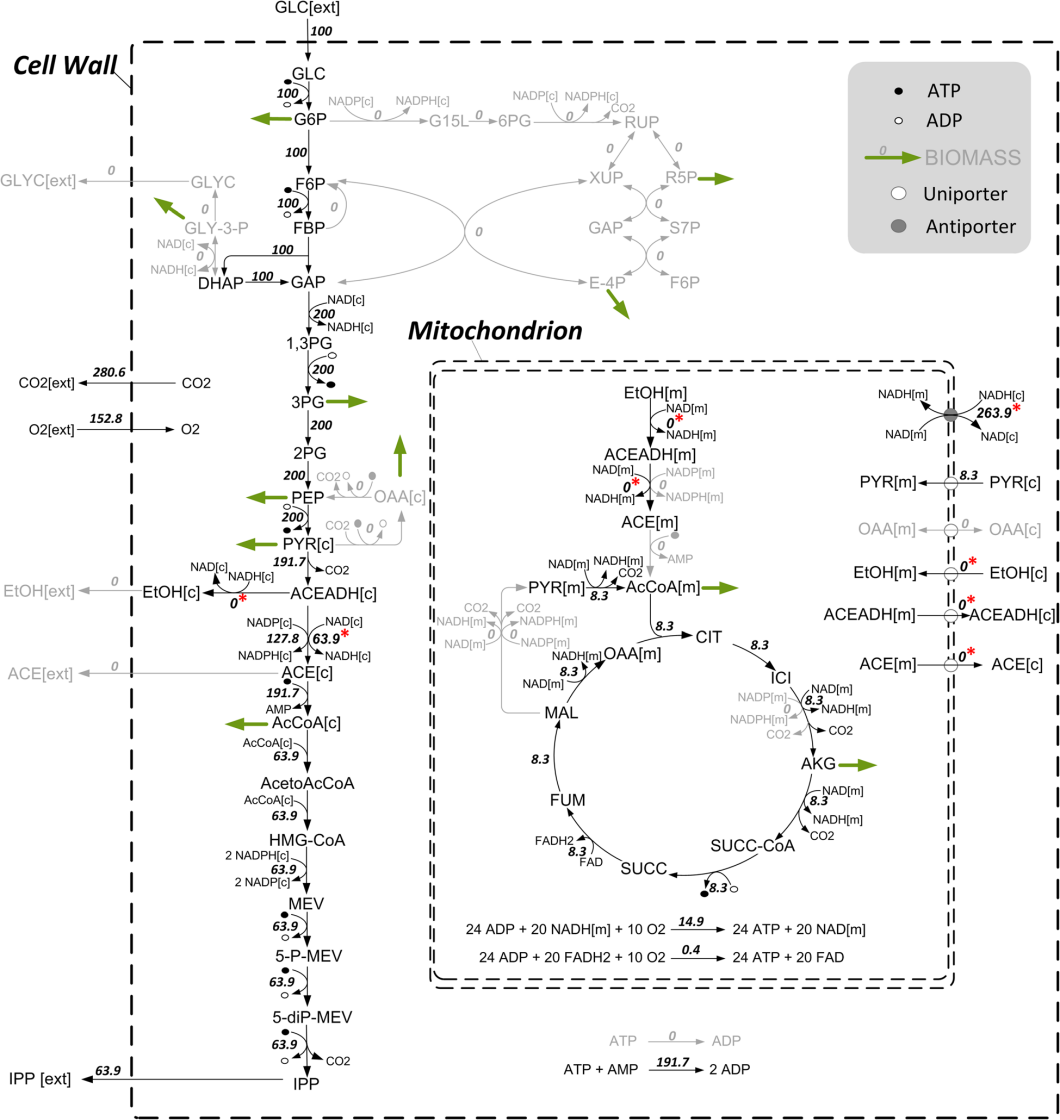
**Optimal flux distribution of *S. cerevisiae. ***Wild type (MVA pathway) on glucose as single carbon source without biomass formation (one out of five modes with maximum theoretical IPP carbon yield). Numbers indicate relative molar fluxes (mmol/(gCDW h), CDW = cell dry weight) normalized to glucose uptake. Grey reactions are not active (flux = 0). Variable fluxes within the 5 optimal modes are marked with an asterisk (*). Bold green arrows indicate biomass precursors.

The differences in carbon stoichiometry of both IPP forming pathways, in energy and redox equivalent requirements as well as in the two hosts’ central carbon metabolisms lead to different theoretical maximal IPP yields. A comparison of the theoretical flux distributions in both organisms provides a detailed picture of the reactions involved and reveals key pathways contributing to maximum IPP production as well as pathways that could be dispensable. Furthermore, they show limitations and provide valuable starting points for metabolic engineering. For *E. coli* and *S. cerevisiae*, two and five elementary modes enable the optimum yield, respectively. In this subsection, main characteristics of these optimal flux distributions are discussed. The flux maps of one particular optimal mode for each case are shown in Figures 3 and 4, respectively.

##### Yield optimal flux distributions in E. coli

In *E. coli*, 3 NADPH as well as 2 ATP are needed for the production of one mol IPP from one mol glucose (see above). In the optimal case, NADPH is provided by a transhydrogenase, an enzyme which transfers reducing equivalents between two nucleotide acceptors to balance the intracellular redox potential (6 NADH + 6 NADP^+^ + 2 ATP = 6 NADPH + 6 NAD^+^ + 2 ADP). However, this leads to an increased requirement for ATP and NADH. Thus, the cell is forced to branch off some carbon flux to the citric acid cycle to generate NADH and ATP as shown in the optimal flux distributions (Figure 3). NADPH is not provided by the oxidative part of the pentose phosphate pathway in this case due to increased carbon loss via CO_2_ which leads to decreased IPP yields. The main flux passes through glycolysis and DXP pathway, while a portion passes pyruvate dehydrogenase complex and citric acid cycle to generate ATP, NADH and NADPH along with respiratory chain and transhydrogenase. The high branch-off leads to a carbon loss and thus to a reduction in the maximum IPP yield from 0.83 (if carbon stoichiometry is considered only) to about 0.67 C-mol/C-mol in the optimal flux distributions (Figure 3, see also Table 1). Some fluxes are flexible within optimal elementary modes namely those of malate dehydrogenase, malate quinone oxidoreductase as well as NADH dehydrogenase II indicating a certain flexibility in redox balancing which does not influence the IPP yield. Optimal IPP production does not involve any by-product formation except CO_2_ and is purely respirative. However, fermentative modes show high product yields as well, up to 0.48 C-mol/C-mol.

The limitation in redox equivalents and energy for high yield IPP production can be validated *in silico* via the introduction of additional artificial ATP, NADH or NADPH sources in the metabolic network model*.* The introduction of an artificial ATP source increases the maximum yield slightly to 0.71, while artificial NADH or NADPH sources lead to the theoretical maximum value of about 0.83 C-mol/C-mol (as discussed in the previous section).

##### Yield optimal flux distributions in S. cerevisiae

In *S. cerevisiae*, 3 NADH and one NADPH are produced via glycolysis while 6 ATP are needed for the production of one mol IPP from 1.5 moles glucose as described above. Generated NADH can be reoxidized via the respiratory chain to produce ATP. However, not enough ATP can be produced due to the stoichiometry of the respiratory chain. Thus, the cell would have a deficiency in energy for high yield IPP production and would be forced to branch off some carbon flux to the citric acid cycle to generate ATP via NADH. This branch-off is shown in the computed optimal flux distributions (Figure 4). The main flux passes glycolysis, pyruvate dehydrogenase bypass and MVA pathway, while a small part is branched off to pyruvate dehydrogenase complex and citric acid cycle in the mitochondria to generate NADH which is then reoxidized via the respiratory chain in order to produce ATP. This branch-off leads to a further carbon loss and thus to a reduction of the IPP yield to about 0.53 C-mol/C-mol in the optimal flux distributions. As shown above, the energy deficiency preventing high yield IPP production of the MVA pathway can be validated via the introduction of an additional artificial ATP or NADH source *in silico* which leads to the theoretical maximum value of about 0.56 C-mol/C-mol. Some fluxes are flexible within optimal elementary modes namely those of mitochondrial and cytosolic alcohol dehydrogenases and NAD^+^-dependent aldehyde dehydrogenases as well as mitochondrial ethanol, acetaldehyde and acetate transport and a redox shuttle indicating a certain flexibility in NADH redox balancing which does not influence the IPP yield. The optimal flux distributions are purely respirative modes. Respiro-fermentative modes (modes that include respiration as well as the formation of fermentation products) show high product yields as well. However, pure fermentative modes are characterized by a very low theoretical maximum product yield of about 0.1 C-mol/C-mol. High ATP requirements of the MVA pathway cannot be delivered by fermentative metabolism. Additionally, the formation of CO_2_, the only side-product, is much higher than in optimal flux distributions in *E. coli* reflecting the high carbon loss in the formation of acetyl-CoA.

Even though theoretical maximal yields differ in *E. coli* and *S. cerevisiae*, the main flux in computed optimal flux distributions at maximum IPP yield is similar. Key pathways are glycolysis and terpenoid pathway and a portion of the flux is directed to citric acid cycle and respiratory chain. Transhydrogenase is active in *E. coli* while pyruvate dehydrogenase bypass is active in yeast to supply NADPH. Dispensable reactions include by-product formation except CO_2_, gluconeogenetic and anaplerotic reactions as well as pentose phosphate pathway if no biomass is produced. Limitations in redox equivalents and energy for high yield IPP production could be validated *in silico.*

#### Exchange of terpenoid pathways

The MVA pathway from *S. cerevisiae* has been functionally introduced in *E. coli* and this strain produced substantially more terpenoid than strains with the native or even engineered DXP pathway [17]. Due to this observation the MVA pathway was introduced *in silico* into *E. coli* to analyze its effects on IPP production. The introduction of the MVA and the removal of the DXP pathway (35,968 EMs) lead to a theoretical maximum value of about 0.56 C-mol/C-mol (which is slightly higher than the value of 0.53 obtained for *S. cerevisiae*). No energy or redox equivalent limitations exist for high yield IPP production. *E. coli* possesses a cytosolic pyruvate dehydrogenase complex instead of a pyruvate dehydrogenase bypass (NADPH source). However, transhydrogenase, Entner-Doudoroff pathway as well as malic enzyme can act as NADPH source. Nevertheless, the sole use of the MVA pathway in *E. coli* is stoichiometrically not efficient as the maximum yield is lower than with the native DXP pathway. The coexistence of both pathways in *E. coli* (44,850 EMs, 1,207 of which that use both pathways simultaneously) leads to a theoretical maximum IPP yield of 0.67 C-mol/C-mol, which is identical to the value if only the DXP pathway is active. However, the theoretical maximum value in an elementary mode, which includes biomass formation, is slightly enhanced to 0.59 C-mol/C-mol. Thus, the coexistence of both pathways is not only beneficial due to the decoupling of the MVA pathway from its native control in yeast but also in terms of stoichiometry.

*In silico*, the introduction of the DXP and the removal of the MVA pathway in *S. cerevisiae* (8,865 EMs) lead to an increase in the theoretical maximum IPP yield to about 0.64 C-mol/C-mol, and 0.57 C-mol/C-mol including biomass formation. The coexistence of both pathways in yeast (11,738 EMs, 276 of which that use both pathways simultaneously) does not lead to a further enhanced IPP yield. Solely the number of elementary modes and thus the flexibility is enhanced. The IPP carbon yield is slightly lower than that one of *E. coli* as yeast does not possess a transhydrogenase to supply NADPH. Hence, pentose phosphate pathway has to deliver NADPH which involves a carbon loss. Pyruvate dehydrogenase bypass can deliver NADPH in the formation of acetyl-CoA; however, acetyl-CoA is not needed for the DXP pathway. The limitation in energy and redox equivalents can be validated *in silico* by the introduction of artificial ATP, NADH and especially NADPH sources in the model, which lead to an enhanced theoretical maximum IPP yield of 0.66-0.8 C-mol/C-mol (see Table 1).

In conclusion, the heterologous introduction of the MVA pathway, in addition to the DXP pathway, into *E. coli* as well as the introduction of the DXP pathway into *S. cerevisiae* are theoretically beneficial for high yield IPP production. Although, the DXP pathway has not been functionally expressed in *S. cerevisiae* yet [13,52], it is a promising target for further research.

#### Comparison of carbon sources

Studies showed that different carbon sources might have a different potential to supply high product yields [8,17,25]. At present, glucose is the most widely used feedstock in bioprocesses [53]. However, other carbon sources are of industrial interest and could serve as substrate for terpenoid production. Molasses consists mainly of sucrose, a disaccharide composed of glucose and fructose, and is a commonly used carbon source [54]. Hemicellulosic hydrolysates of agricultural by-products like sugarcane bagasse or wood consist mainly of xylose and glucose as well as other sugars like galactose [55] and could be a cheap alternative carbon source for bioprocesses [56]. Pure glycerol is used in different industries too and crude glycerol could be a cheap alternative as it is a by-product from biodiesel production with decreasing price [53,57]. Thus, EMA was further performed for glycerol for *E. coli.* Moreover, the carbon sources galactose, fructose and xylose were analyzed for *S. cerevisiae* as well as glycerol and ethanol because high terpenoid yields have been described with this substrate [8]. The metabolic networks of the non-fermentable carbon sources ethanol and glycerol involve the glyoxylate cycle and an increased number of mitochondrial shuttles in yeast.

##### Alternative substrate for E. coli

The maximum IPP yield in *E. coli* on the non-fermentable carbon source glycerol purely based on carbon stoichiometry (ATP and NAD(P)H considered as external metabolites) is identical to that on glucose (0.83 C-mol/C-mol). The overall stoichiometry for the formation of one mol IPP from glycerol is given by equation 5.

(5) $$\begin{array}{ll} \;2 & \text{GLYC}+2\;\text{ATP}+\text{NADPH}+{\text{NAD}}^{+}\\ & =\text{IPP}+{\text{CO}}_{2}+2\;\text{ADP}+{\text{NADP}}^{+}+\text{NADH} \end{array}$$

The carbon loss for the formation of IPP from glycerol is identical to glucose. However, less NADPH is required as it is supplied by glycerol-3-phosphate dehydrogenase. If the balances of ATP, NADH and NADPH are taken into account, the yields for *E. coli* and the native DXP pathway on glycerol (26,160 EMs) were enhanced in comparison to glucose *in silico* (0.78 C-mol/C-mol at zero growth and 0.77 C-mol/C-mol in an elementary mode that includes biomass formation). Yields were slightly lower for the heterologous MVA pathway (0.56 C-mol/C-mol at zero growth and 0.45 C-mol/C-mol including biomass formation). Thus, glycerol and the native DXP pathway have the highest potential for *E. coli* to support high IPP production. The main flux in optimal flux distributions passes lower part of glycolysis and DXP pathway, while only a small portion is diverted to citric acid cycle, respiratory chain and transhydrogenase to generate NADPH, NADH and ATP. The limitation in energy and redox equivalents for high yield IPP production can be validated *in silico* by the introduction of artificial ATP, NADH or NADPH sources in the model, which lead to the maximum IPP yield of 0.83 C-mol/C-mol (see Table 1).

##### Alternative substrates for S. cerevisiae

The carbon as well as the overall stoichiometry for the formation of one mol IPP from galactose or fructose is identical to glucose (see equation 4). Accordingly, the numbers of elementary modes as well as the IPP and biomass yields with galactose and fructose are identical to those with glucose. A heterologous pathway has to be introduced into *S. cerevisiae* for xylose as wild type strains are not able to grow on the pentose xylose. Two different pathways have been used for ethanol production based on xylose with yeast: the xylose isomerase (XI) pathway as well as the xylose reductase and xylitol dehydrogenase (XR-XDH) pathway [58]. The carbon stoichiometry for the formation of one mol IPP from xylose is identical to glucose (maximum 0.56 C-mol/C-mol). Likewise, the overall stoichiometry is identical to the one on glucose for the XI pathway except that 1.8 mol xylose (XYL) is needed per mol IPP (equation 6).

(6) $$\begin{array}{ll} \;1.8 & \text{XYL}+6\;\text{ATP}+{\text{NADP}}^{+}+3\;{\text{NAD}}^{+}\\ & =\text{IPP}+4\;{\text{CO}}_{2}+6\;\text{ADP}+\text{NADPH}+3\;\text{NADH} \end{array}$$

The overall stoichiometry is identical for the XR-XDH pathway if xylose reductase uses NADH (as xylitol dehydrogenase delivers NADH) and slightly changes if the enzyme uses NADPH. The theoretical maximal IPP and biomass yields are identical to the values on glucose for both the XI (6,330 EMs) and the XR-XDH pathway (16,911 EMs). However, the IPP yield including biomass formation is enhanced to 0.51 C-mol/C-mol with the XR-XDH pathway. The number of elementary modes and thus the flexibility of the metabolic network is greatly enhanced if the XR-XDH pathway is chosen. The optimal flux distributions were basically identical to those on glucose except that xylose enters glycolysis via the non-oxidative part of pentose phosphate pathway.

The maximum IPP yield on the non-fermentable carbon source glycerol (GLYC) purely based on carbon stoichiometry (ignoring energy and redox equivalents) is with 0.56 C-mol/C-mol identical to glucose. However, the overall stoichiometry is slightly modified (equation 7).

(7) $$\begin{array}{ll} \;3 & \text{GLYC}+3\;\text{ATP}+{\text{NADP}}^{+}+6\;{\text{NAD}}^{+}\\ & =\text{IPP}+4\;{\text{CO}}_{2}+3\;\text{ADP}+\text{NADPH}+6\;\text{NADH} \end{array}$$

Less energy is required and more NADH is delivered for the production of one mol IPP from glycerol. Glycerol is channeled into glycolysis via glyceraldehyde-3-phosphate and the main flux passes lower part of glycolysis, pyruvate dehydrogenase bypass and the MVA pathway. The carbon flux is not diverted to citric acid cycle as enough energy is produced via NADH and respiratory chain. Thus, the maximum IPP yield of 0.56 C-mol/C-mol is achieved, which is identical to the maximum IPP yield based on carbon stoichiometry only. No limitations in energy or redox equivalent requirements thus exist for high yield IPP production. The optimal flux distributions further reveal that pentose phosphate pathway, side product formation, the first part of citric acid cycle as well as glyoxylate cycle are not active. Some fluxes are flexible namely those of alcohol dehydrogenases, NAD^+^-dependent aldehyde dehydrogenases, second part of citric acid cycle as well as mitochondrial transport systems indicating a certain flexibility in NADH redox balancing which does not influence the IPP yield. The very high number of elementary modes (2,648,133 EMs) implies an increased redundancy and thus network flexibility and is due to the activity of the glyoxylate cycle. The glyoxylate cycle showed only a weak activity in yeast cells grown on glycerol in our experiments (data not shown). If the cycle is excluded from the computations, the number of elementary modes decreases (5,377 EMs) but the theoretical maximum IPP yield remains 0.56 C-mol/C-mol. Nevertheless, the IPP yield including biomass formation is much higher if the cycle is included (0.47 C-mol/C-mol versus 0.35 C-mol/C-mol) indicating that an active glyoxylate cycle could be of benefit.

The maximum IPP yield on the non-fermentable carbon source ethanol purely based on carbon stoichiometry is with 0.83 C-mol/C-mol as high as the IPP yield in *E. coli*. The overall stoichiometry for the formation of one mol IPP from ethanol (EtOH) is given by equation 8.

(8) $$\begin{array}{ll} \;3 & \text{EtOH}+9\;\text{ATP}+{\text{NADP}}^{+}+3\;{\text{NAD}}^{+}\\ & =\text{IPP}+{\text{CO}}_{2}+9\;\text{ADP}+\text{NADPH}+3\;\text{NADH} \end{array}$$

In contrast to glucose or glycerol as the carbon source, the formation of IPP from ethanol requires more energy but does not involve an additional carbon loss via CO_2_ (except the carbon loss in the terpenoid pathway itself). In fact, similarly to glycerol and *E. coli*, ethanol (2,874,235 EMs) reveals an increased theoretical maximum product yield of 0.68 C-mol/C-mol at zero growth as well as 0.59 C-mol/C-mol including biomass formation for the MVA pathway. The high number of elementary modes is again due to the activity of the glyoxylate cycle. The optimal flux distributions of yeast (without biomass formation) with only the MVA pathway reveal that the glycolysis, gluconeogenesis, pentose phosphate pathway and side product formation are not active. The flux passes pyruvate dehydrogenase bypass, glyoxylate cycle as well as citric acid cycle and respiratory chain to generate ATP. The energy deficiency preventing high IPP yields can be validated *in silico* by introducing artificial ATP or NADH sources which lead to the maximum yield of 0.83 C-mol/C-mol. As expected from these computations, the sesquiterpenoid yield of the same yeast strain was increased significantly *in vivo* by changing the carbon source from glucose (0.0111 C-mol/C-mol) to ethanol (0.1867 C-mol/C-mol) [8].

The introduction of the DXP pathway *in silico* and the growth on galactose, fructose or xylose lead to similar yields to those on glucose, except that the theoretical maximal IPP yields including biomass formation are slightly increased on xylose. With ethanol as substrate, the heterologous DXP pathway shows a lower potential compared to the native MVA pathway, with yields of 0.63 C-mol/C-mol at zero growth and 0.57 C-mol/C-mol including biomass formation. Lower yields are due to energy-consuming gluconeogenetic reactions which are essential to provide the precursors of the DXP pathway from ethanol. Nevertheless, the introduction of the DXP pathway could be beneficial for growth on glycerol. The theoretical maximum IPP yield is enhanced to 0.67 C-mol/C-mol at zero growth and 0.6 C-mol/C-mol including biomass formation. The cells would have a limitation in NADPH for high yield IPP production, which can be validated via the introduction of an artificial NADPH source leading to the maximum value of 0.83 C-mol/C-mol.

To summarize this subsection, sugars like glucose, galactose, fructose and even xylose lead to identical or similar theoretical maximal IPP yields in *S. cerevisiae* while the non-fermentable carbon sources ethanol and glycerol show the highest potential with respect to yield and network flexibility. Similarly, glycerol shows the highest potential in *E. coli* to supply IPP in high yields.

#### Comparison of theoretical yields with experimental yields reported in literature

A comparison of computed theoretical maximal IPP yields with terpenoid yields published in literature is shown in Table 2. The experimental carbon yields are still very low, even though strains used in these studies had been genetically modified within the respective terpenoid pathway. This highlights the tremendous potential of yield improvement. The terpenoid yield for yeast on ethanol was quite high in a study [8] indicating that the capacity of the terpene synthase or the terpenoid pathway (MVA) are not the only limiting factors but that the flux into these pathways is a limiting factor on other carbon sources like glucose. This shows that, besides the already very well-studied modifications within the terpenoid pathway, genetic modifications within the central carbon metabolism are indispensable to efficiently redirect the carbon flux to the terpenoid pathway (‘push and pull’ strategy).

**Table 2:** Comparison of theoretical maximal IPP yields with published terpenoid yields (given as C-mol/C-mol)

**Organism**	**Pathway**	**Carbon source**	**Maximum IPP yield (this study)**	**Experimental IPP yield estimated from published yield**
*E. coli*	DXP	glycerol+	0.79	0.04 [59]
*E. coli*	DXP	glycerol+	0.79	0.06 [14]
*E. coli*	DXP + MVA	glycerol+	0.79	0.04 [60]
*S. cerevisiae*	MVA	glucose+	0.53	0.003 [61]
*S. cerevisiae*	MVA	glucose+	0.53	0.00006 [62]
*S. cerevisiae*	MVA	galactose	0.53	0.004 [30]
*S. cerevisiae*	MVA	glucose	0.53	0.0052 [31]
*S. cerevisiae*	MVA	glucose	0.53	0.01 [8]
*S. cerevisiae*	MVA	glucose&ethanol	0.53/0.68	0.06 [8]
*S. cerevisiae*	MVA	ethanol	0.68	0.19 [8]

Experimental yields of sesquiterpenoids are partly estimated and converted into IPP yields (only to that part of IPP that has been converted to the respective terpenoid); + indicates complex media.

### Target identification for metabolic engineering

#### Identification of overexpression targets

The identified limitations in the generation of sufficient energy and redox equivalents for high yield IPP production with both organisms can be used as starting point for metabolic engineering. Evidently, a reasonable approach would be to implement heterologous enzymes that are more efficient with respect to NAD(P)H or ATP consumption.

For *E. coli*, an increase in the maximum yield could be accomplished by the introduction of alternative pathways with less energy requirements or additional NAD(P)H sources. For instance, an additional heterologous NADP^+^-dependent glyceraldehyde-3-phosphate dehydrogenase enhances the theoretical maximum IPP yield to 0.71 C-mol/C-mol on glucose and to 0.83 C-mol/C-mol on glycerol.

For *S. cerevisiae*, an increase in the maximum IPP yield could be accomplished by the introduction of alternative pathways with less energy requirements. *S. cerevisiae* possesses a mitochondrial pyruvate dehydrogenase complex and a cytosolic pyruvate dehydrogenase bypass to produce acetyl-CoA. Solely acetyl-CoA produced via the bypass, which requires ATP, can be used for terpenoid production since acetyl-CoA cannot be exported from the mitochondria. In contrast, *E. coli* possesses a cytosolic pyruvate dehydrogenase complex, which does not require energy in the form of ATP. *In silico*, a heterologous introduction of cytosolic pyruvate dehydrogenase complex from *E. coli* into *S. cerevisiae* leads to the theoretical maximum IPP yield of 0.56 C-mol/C-mol and to a mode enabling biomass formation coupled to an increased IPP yield of 0.51 C-mol/C-mol on glucose. A cytosolic pyruvate dehydrogenase complex has to our knowledge not been introduced into yeast with the aim of enhancing terpenoid production. Though, a patent application indicates that the complex of *E. coli* (*lpdA*, *aceA*, *aceF*) might increase the cytosolic acetyl-CoA pool for butanol production [63].

A different approach would be to transfer the native MVA pathway from *S. cerevisiae* into the mitochondria. Likewise, on glucose, the theoretical maximum IPP yield at zero growth is enhanced to 0.56 C-mol/C-mol and the IPP yield including biomass formation is enhanced to 0.51 C-mol/C-mol, if only the MVA in the mitochondria is active, or to 0.56 and 0.53 C-mol/C-mol if the MVA is active in the cytosol and the mitochondria. The mitochondrial IPP and DMAPP as well as the FPP pool have been harnessed successfully for terpenoid production [10]. However, the whole MVA pathway has not been transferred to the mitochondria. The advantage of this approach would be that the mitochondrial acetyl-CoA pool, which is formed via the pyruvate dehydrogenase complex that does not require ATP, would be used.

Alternatively, an ATP-citrate-lyase can be expressed heterologously in the cytosol. The enzyme forms cytosolic acetyl-CoA from citrate that has been exported from the mitochondria and thus circumvents pyruvate dehydrogenase bypass. The reaction is accompanied by the conversion of one mol ATP to ADP per acetyl-CoA, while the cytosolic pyruvate dehydrogenase bypass converts in the net one mol ATP to AMP. Thus, ATP-citrate-lyase is more energy-efficient and thus promising. The theoretical maximum IPP yield at zero growth is indeed enhanced to 0.55 C-mol/C-mol. Moreover, a patent application indicates that this approach might be promising [64].

If the DXP pathway is introduced into yeast, a heterologous soluble transhydrogenase leads to a higher theoretical maximum IPP yield of 0.67 C-mol/C-mol on glucose. However, the introduction of a heterologous soluble transhydrogenase from *Azotobacter vinelandii* as well as the introduction of a membrane-bound enzyme from *E. coli* into *S. cerevisiae* led to a reduction of the NADPH pool including an increase in glycerol formation [65-67], which is the opposite of the desired effect. Though, the introduction of a heterologous NADP^+^-dependent glyceraldehyde-3-phosphate dehydrogenase leads *in silico* to the same improvement in IPP yield on glucose and to a yield of 0.76 C-mol/C-mol on glycerol.

#### Identification of knockout strategies

The constrained minimal cut sets (cMCSs) approach allows the identification of all possible knockout combinations that eliminate undesired (low-yield) elementary modes and keep desired modes, which (i) have a specified minimum product yield and (ii) allow at least some biomass formation [40]. Hence, a selection pressure imposed by gene deletions forces the cell to produce a predefined minimum IPP yield to be able to grow. However, optimal biological functions are acquired through evolutionary processes. For *in vivo* validation, it has been shown that adaptive evolution is essential to improve production capabilities from sub-optimal metabolic states to optimal ones as predicted from *in silico* analysis [68,69].

In this study, cMCSs are used to identify combinations of gene deletions for both *E. coli* and *S. cerevisiae* to present new intervention targets for metabolic engineering.

##### cMCSs for E. coli

**Figure 5:**
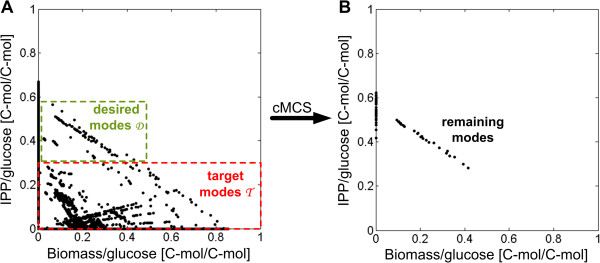
**Concept of cMCSs illustrated with an example of *E. coli*. A**: Solution space formed by the elementary modes of the wild type network of *E. coli* (36,590 EMs; compare Figure 2). The biomass yield on glucose is plotted against the IPP yield on glucose [C-mol/C-mol] of all computed elementary modes. The deletion task is defined by collecting all modes exhibiting an IPP yield lower than 0.25 C-mol/C-mol in the set of target modes $\tilde{T}$ (33,087 EMs). The set of desired modes $\tilde{D}$ comprises all EMs having (i) a minimum IPP yield of 0.25 C-mol/C-mol; and (ii) a simultaneous biomass yield strictly higher than zero (268 EMs). **B**: Example of one specific cMCS. The *in silico* knockout of transhydrogenase and phosphoglycerate mutase (strategy 2 in Table 3) leads to a reduction of elementary modes. Remaining elementary modes (191) display a minimum IPP yield of 0.28 and a maximum of 0.62 C-mol/C-mol as well as a maximum biomass yield of 0.41 C-mol/C-mol on glucose. In those modes, biomass formation is coupled to a minimum yield of IPP production.

Constrained minimal cut sets were computed for the *E. coli* wild type metabolic network on glucose (36,590 EMs) as well as glycerol as carbon source (26,160 elementary modes). For glucose, the deletion task was defined by collecting all elementary modes exhibiting an IPP yield lower than 0.25 C-mol/C-mol in the set of target modes $\tilde{T}$ (33,087 EMs). The set of desired modes $\tilde{D}$ comprises all EMs having (i) a minimum IPP yield of 0.25 C-mol/C-mol; and (ii) a simultaneous biomass yield necessarily higher than zero (268 modes) (see Figure 5). When at least one EM of the set of $\tilde{D}$ should be preserved (*n* = 1; see Methods), a total of 488 cMCSs accomplishing the specified engineering goal were computed. Cut sets including diffusion processes or parts of respiratory chain were excluded from further considerations due to poor feasibility leaving 28 cMCSs with two or four interventions (see Table 3). One group of cut sets forces a coupling of IPP production with biomass formation via redox equivalents. This is achieved by preventing oxygen uptake and thus respiration as well as side product formation and reactions within citric acid cycle that can reoxidize NADH (see Table 3). Hence, NADH has to be reoxidized via transhydrogenase which leads to excess NADPH production. Excess NADPH is then reoxidized via the DXP pathway. Applying these cut sets, the remaining elementary modes are anaerobic, exhibit a minimum IPP carbon yield of 0.38 and the maximum anaerobic IPP yield of 0.48. However, the maximum possible biomass carbon yield is very low. A second group of cut sets includes the knockout of the lower part of glycolysis to force the cell to use the Entner-Doudoroff pathway instead, which results in less ATP but more NADPH. The cell cannot reoxidize excess NADPH by the additional knockout of transhydrogenase. Excess NADPH is then reoxidized via the DXP pathway. Remaining elementary modes are aerobic, exhibit a slightly lower minimum IPP carbon yield but have a much higher maximum biomass carbon yield than the anaerobic modes.

**Table 3:** **cMCSs for *E. coli: *number of remaining elementary modes (EMs), minimum and maximum IPP yield as well as maximum biomass yield on glucose or glycerol [C-mol/C-mol] for different knockout strategies**

**cMCS knockout strategy**	**EMs**	**Min. IPP yield**	**Max. IPP yield**	**Max. biomass yield**
**Wild type on glucose**	36,590	-	0.67	0.85
**Strategy 1**: O_2_ uptake + {lactate ex OR lactate formation} + {ethanol ex OR ethanol formation OR acetaldehyde formation} + {succinate ex OR succinate OR malate DH OR fumarate hydratase}	60-86	0.38	0.48	0.08
**Strategy 2**: transhydrogenase + {phosphoglycerate mutase OR enolase}	191	0.28	0.62	0.41
**Strategy 3**: transhydrogenase + {GAP DH OR phosphoglycerate kinase}	191	0.35	0.62	0.32
**Wild type on glycerol**	26,160	-	0.78	0.99
**Strategy 1**: transhydrogenase + pyruvate kinase + pyruvate DH complex + ribulose-phosphate 3-epimerase + acetate formation + glyoxylate shunt + {succinate DH OR fumarate hydratase}	22	0.38	0.68	0.40

DH = dehydrogenase; ex = excretion.

Another cut set included glucose-6-phosphate-isomerase, transhydrogenase, Entner-Doudoroff pathway as well as one specific part of respiratory chain. The cMCS is not shown in Table 3 due to poor feasibility of the last target. However, it serves as an example because it has been implemented. The coupling is again due to excess NADPH production. The knockout of glucose-6-phosphate-isomerase (*∆pgi*) has been done in *E. coli in vivo* and led to an increased flux through pentose phosphate pathway and thus to excess NADPH formation. However, an additional knockout of transhydrogenase (*∆pgi ∆udhA*) was lethal due to NADPH imbalance in this wild type strain [70]. This example illustrates that the principle of the method is effective but regulatory constraints may interfere with proposed knockout strategies. One can assume that the overexpression of a terpenoid synthase together with the DXP pathway as a sink and eventually adaptive evolution could balance the excess NADPH production via a ‘push and pull’ strategy and lead to the predicted coupling.

For glycerol, the deletion task was set up by (i) defining $\tilde{T}$ as the set of elementary modes exhibiting an IPP yield of not more than 0.33 C-mol/C-mol (22,801 EMs); (ii) defining $\tilde{D}$ as all EMs with a minimum IPP yield of 0.33 C-mol/C-mol and a simultaneous biomass formation strictly higher than zero (570 EMs); and (iii) demanding that at least one EM of $\tilde{D}$ is preserved (*n* = 1). A total of 769 cMCSs were computed, while cut sets that cannot be realized in practice (including diffusion processes, parts of respiratory chain, ATP maintenance etc.) were excluded from further considerations leaving only 8 cMCSs with eight interventions each (see Table 3). Feasible minimal cut sets include the knockout of transhydrogenase, pyruvate kinase, pyruvate dehydrogenase complex, ribulose-phosphate 3-epimerase, acetate formation, glyoxylate shunt and citric acid cycle (succinate dehydrogenase or fumarate hydratase). A minimum IPP carbon yield of 0.38 C-mol/C-mol can be guaranteed but only two of the remaining elementary modes enable biomass formation.

The presented minimal cut set strategies can also be applied to *E. coli* strains carrying the additional MVA pathway. The IPP and biomass yields of remaining elementary modes are only slightly modified (results not shown).

The cMCSs approach points to new and promising combinations of metabolic engineering targets. The predicted minimal IPP yields are much higher than the yields that have been published in literature so far. Nevertheless, kinetics and regulation, especially of the DXP pathway, may impose additional challenges to be solved in order to achieve high terpenoid yields with *E. coli*.

##### cMCSs for S. cerevisiae

Constrained minimal cut sets were computed based on the wild type metabolic network with glucose as carbon source (9,844 EMs). Modes exhibiting an IPP yield of not more than 0.25 C-mol/C-mol were collected in the set of target modes $\tilde{T}$ (9,453 EMs). All EMs exhibiting (i) an IPP yield of not less than 0.25 C-mol/C-mol; and (ii) a concurrent biomass yield strictly higher than zero were defined as desired modes $\tilde{D}$ (322 EMs). While preserving at least one of the desired modes (*n* = 1), a total of 407 cMCSs were computed. Cut sets including mitochondrial transport systems, diffusion, ATP maintenance etc. were excluded from further considerations leaving 8 feasible cMCSs with three to six interventions (Table 4). All remaining cMCS require blocking of acetate secretion and either of ethanol secretion or of all alcohol dehydrogenases (cytosolic and mitochondrial). The prevention of acetate and ethanol production is crucial as the engineering goal cannot be accomplished without those two targets. An additional target is the partial knockout of citric acid cycle after α-ketoglutarate, namely α-ketoglutarate dehydrogenase or succinyl-CoA ligase (succinate dehydrogenase or fumarate hydratase would be an alternative as well but were not considered due to possible succinate secretion). Alternatively malate dehydrogenase and malic enzyme can be deleted for a partial knockout of citric acid cycle. A second alternative is malate dehydrogenase, NAD^+^-dependent isocitrate dehydrogenase as well as pyruvate dehydrogenase complex.

**Table 4:** **cMCSs for *S. cerevisiae*: number of remaining elementary modes (EMs), minimum and maximum IPP carbon yield as well as maximum biomass carbon yield on glucose [C-mol/C-mol] for different knockout strategies**

**cMCS knockout strategy**	**EMs**	**Min. IPP yield**	**Max. IPP yield**	**Max. biomass yield**
**Wild type**	9,844	-	0.53	0.80
**Strategy 1**: acetate ex + {ethanol ex OR alcohol DH} + {α-ketoglutarate DH OR succinyl-CoA ligase}	142/48	0.33	0.53	0.29
**Strategy 2**: acetate ex + {ethanol ex OR alcohol DH} + malate DH + malic enzyme	60/21	0.33	0.53	0.29
**Strategy 3**: acetate ex + {ethanol ex OR alcohol DH} + malate DH + NAD^+^-dependent isocitrate DH + pyruvate DH complex	51/19	0.27	0.53	0.37
**Wild type plus DXP pathway**	11,738	-	0.64	0.80
**Strategy 1**: glucose-6-phosphate-isomerase	631	0.37	0.63	0.35

DH = dehydrogenase; ex = excretion.

Possible knockout combinations were simulated and Table 4 shows the number of remaining elementary modes and the minimal and maximal IPP and biomass carbon yields when applying the respective cMCS. All minimal cut sets lead to a reduced maximum biomass yield and a reduced number of elementary modes. However, a certain flexibility is preserved. The IPP carbon yield can reach the maximum value of the wild type, and a minimum value of 0.27 or 0.33 is computed. Due to the partial disruption of citric acid cycle the cell is not able to convert glucose completely to CO_2_. Hence, the cell is forced to secrete products like ethanol or acetate. The repression of acetate and ethanol excretion forces the cell to find another sink like IPP. Targets identified for glucose can also be applicable to other sugars like galactose or fructose.

The knockout of succinyl-CoA ligase (∆*LSC1*) was implemented in *S. cerevisiae* and an enhanced acetate secretion was observed [71]. This indicates that the principle of the method is effective even though acetate is produced instead of channeling the flux to acetyl-CoA, the precursor of the MVA pathway. Nevertheless, acetate secretion can be lowered by the overexpression of acetyl-CoA synthetase [48]. Moreover, an overexpression of the MVA pathway together with a terpenoid synthase might channel the flux to IPP via the ‘push and pull’ strategy.

cMCSs including a heterologous DXP pathway are basically identical to those of the wild type. Thus, presented strategies can also be applied to a strain carrying an additional DXP pathway if the pathway was active *in vivo*. However, the minimal IPP yields are unchanged and thus the heterologous DXP pathway would be of no benefit. Nevertheless, one additional interesting intervention strategy was computed. The single knockout of glucose-6-phosphate-isomerase leads to a coupling of biomass formation to a minimal IPP yield of 0.37 C-mol/C-mol. The knockout of glucose-6-phosphate-isomerase forces the cell to direct the flux completely to pentose phosphate pathway, which leads to excess NADPH production. Wild type yeast cells are not able to compensate excess NADPH as they do not possess a transhydrogenase. Further, NADH dehydrogenases (Ndi1p, Nde1p, Nde2p) do not oxidize NADPH and malic enzyme reaction seems to be irreversible *in vivo*[72,73]. Yeast cells are not able to grow on glucose after the knockout of *PGI1* gene encoding glucose-6-phosphate-isomerase [74] and no elementary modes could be computed for the wild type network including only the MVA pathway with the single knockout of glucose-6-phosphate-isomerase. Nevertheless, an active DXP pathway could serve as a NADPH sink as it requires more NADPH and less ATP per mol IPP than the MVA pathway.

Minimal cut sets were also computed for the metabolic network with xylose as carbon source including either the XI or the XR-XDH pathway together with the MVA pathway. Identified knockout targets as well as yields after the implementation of a minimal cut set for the XI pathway are identical to those of glucose. Knockout targets for the XR-XDH pathway are different. Minimal cut sets only exist with an additional target, ribulose-5-phosphate-3-epimerase in pentose phosphate pathway. Cut sets including pyruvate dehydrogenase complex do not exist. Moreover, possible IPP yields after the implementation of a minimal cut set are slightly different and can vary between 0.30 and 0.50 C-mol/C-mol.

To our knowledge, a disruption of citric acid cycle has not been done with the aim to enhance terpenoid production. Predicted minimal IPP yields are much higher than yields that have been achieved in literature so far. Thus, targets identified by the minimal cut sets approach are completely new and promising for an enhanced terpenoid yield on sugars with *S. cerevisiae*. Moreover, the knockout of glucose-6-phosphate-isomerase appears as a very promising strategy to enhance terpenoid production if the DXP pathway can be functionally expressed in yeast.

## Conclusions

The use and optimization of microorganisms for an ecologically and economically efficient production of plant terpenoids is a contemporary issue in academic research as well as industry. The most widely used model organisms *E. coli* and *S. cerevisiae* as well as their respective terpenoid pathway, heterologous enzymes and different carbon sources have been analyzed *in silico* in this study with respect to their potential as terpenoid factories. The DXP pathway of *E. coli* has a tremendously higher potential for high IPP yields (nearly 50% higher) than the MVA pathway of yeast with respect to carbon stoichiometry due to their different precursors. However, kinetics, regulation and a balanced overexpression have to be considered for *in vivo* production. Further research should focus on the optimization of the DXP pathway to tap its full potential. *E. coli* wild type shows a higher theoretical potential to supply IPP in high yields than *S. cerevisiae* wild type. However, the theoretical maximal yields can be enhanced in both organisms by introducing heterologous enzymes or whole pathways or by simply changing the carbon source. Moreover, new constrained minimal cut set strategies can be applied to both hosts to enforce a coupling of growth to a minimum IPP yield which is higher than any yield published in scientific literature so far.

## Methods

### Elementary modes and minimal cut sets

Consider a given metabolic reaction network with q × p stoichiometric matrix **N** (with q reactions and p metabolites) and a set *Irrev* of irreversible reactions. The set of steady-state flux vectors **r** form the convex (flux) cone reads:

$$F=\left\{r\in R^{q}|\;\mathrm{Nr}=0,\;r_{i}\geq 0\;\forall i\in \mathrm{Irrev}\;\right\}\;$$

An Elementary Mode is a non-decomposable vector **e** ∈ ***F*** , i.e. for every vector **r** ∈ ***F***, **r** ≠ 0, one has supp(**e**) ⊆ supp(**r**), where the support of a vector is defined as the index set of all reactions carrying a non-zero flux (supp(**r**) = {*i* | *r*_*i*_ ≠ 0}). EMs correspond to minimal functional units (pathways or cycles) of a metabolic network and are useful to study various functional network properties [34,36].

In contrast, a minimal cut set (MCS) is a (support-) minimal set ***C*** of reactions, the removal of which will block (disrupt) a given set ***M*** of target flux vectors, ***M*** ⊆ ***F***[75,76]:

$$\left\{r\in F|\;r_{i}=0,\forall i\in C\right\}\cap M=\emptyset$$

One drawback of the original definition of MCSs is that besides the targeted functionalities some desired functionalities can be lost as well. Therefore, a generalization to constrained MCSs was introduced [40] which can be described as follows (in the following, EMs are represented by their support, i.e. by the set of reactions carrying a non-zero flux in the EM):

1) Define a set of target modes $\tilde{T}$ (where each target mode **t** is represented by its support ***T*** = *supp*(**t**)).

2) Define a set of desired modes $\tilde{D}$ (where each desired mode **d** is represented by its support ***D*** = *supp*(**d**)).

3) Specify a number *n* quantifying the minimal number of EMs from $\tilde{D}$ that must be kept in the network, i.e. a cMCS C fulfills: $\forall T\in \tilde{T}:C\cap T\neq \emptyset \wedge |\left\{D\in \tilde{D}|D\cap C=\emptyset \right\}|\geq n$.

cMCSs provide a flexible and convenient approach for formulating and solving intervention problems. The approach also allows one to compute interventions that lead to robust coupling of product and biomass formation (coupling even for suboptimal growth). To compute the cMCSs, we applied an adapted Berge algorithm (allowing the computation of cMCSs as minimal hitting sets of $\tilde{T}$ subject to $\tilde{D}$ and *n*), implemented in the software package *CellNetAnalyzer*[77].

### Metabolic networks

A comprehensive analysis of the complete set of elementary modes is still infeasible with genome-scale metabolic models. Therefore, stoichiometric models of the central metabolism of *E. coli* and *S. cerevisiae* were built up manually. The basic glucose network for *E. coli* consists of 65 reactions, of which 21 are reversible, and 50 metabolites (12 external). For *S. cerevisiae* the basic glucose network comprises 69 reactions, thereof 30 reversible, and 60 metabolites (8 external). The P/O-ratios (mol ATP produced per 0.5 mol O_2_ reduced) representing the efficiency of ATP production are assumed to be 1.2 for *S. cerevisiae*[78,79] and for *E. coli* 2 from NADH and 1 from quinol [39,43,80]. The metabolic networks of *S. cerevisiae* and *E. coli* have been constructed considering the current knowledge from genome-scale models and literature data [81-86]. The most important reactions are depicted in Figures 3 and 4, respectively. Details of the models (including the complete list of enzymes, genes and considered literature) are given in Additional file 1.

## Abbreviations

AcCoA: Acetyl-CoA; ACL: ATP-citrate-lyase; CDW: Cell dry weight; cMCS: Constrained minimal cut set; DH: Dehydrogenase; DMAPP: Dimethylallyl diphosphate; DXP: 1-deoxy-D-xylulose 5-phosphate; EM: Elementary mode; EMA: Elementary mode analysis; EtOH: Ethanol; Ex: Excretion; FPP: Farnesyl diphosphate; GAP: Glyceraldehyde-3-phosphate; GLC: Glucose; GLYC: Glycerol; IPP: Isopentenyl diphosphate; MCS: Minimal cut set; MEP: 2C-methyl-D-erythriol 4-phosphate; MVA: Mevalonate; PDH: Cytosolic pyruvate dehydrogenase complex; PYR: Pyruvate; TH: Soluble and energy-independent transhydrogenase; XI: Xylose isomerase; XR-XDH: Xylose reductase and xylitol dehydrogenase; XYL: Xylose.

## Competing interests

The authors declare that they have no competing interests.

## Authors’ contributions

EG did all the computations and interpretations. OH and SK supported the constrained minimal cut sets computations. EG and VS designed the study. EG, VS, OH and SK wrote and drafted the manuscript. OK supervised the research. VS conceived of the study and supervised the research. All authors read and approved the final manuscript.

## Supplementary Material

Additional file 1 **Metabolic networks.** Metabolic networks of *S. cerevisiae* and *E. coli* including the complete list of genes, heterologous enzymes, alternative carbon sources and considered literature. Click here for file
