# Supplementary material for: In silico profiling of Escherichia coli and Saccharomyces cerevisiae as terpenoid factories
Source: Microb Cell Fact. 2013 Sep 23;12:84. doi: 10.1186/1475-2859-12-84 (PMC3852115; doi:10.1186/1475-2859-12-84)
Supplement: Additional file 1 — Metabolic networks. Metabolic networks of S. cerevisiae and E. coli including the complete list of genes, heterologous enzymes, alternative carbon sources and considered literature. [file 1475-2859-12-84-S1.docx]

**Additional file 1: Metabolic networks**

The metabolic networks of *S. cerevisiae* and *E. coli* have been constructed considering the current knowledge from genome-scale models and literature [[1-6](#_ENREF_1)] as well as the literature cited in the following tables. Since H_2_O and P_i_ are assumed to be ubiquitous and CoA-SH is part of a conservation relation, these species are not considered explicitly. For *S. cerevisiae,* reactions and metabolites are compartmentalized between cytosolic [c] and mitochondrial [m] compartments.

***S. cerevisiae***

**Wild type network on glucose**

| **Enzyme/Description** | **Gene (systematic name)** | **Gene (standard name)** | **Reaction** |
| --- | --- | --- | --- |
| **Transport** | | | |
| Transport: hexose transporter (*HXT 1-11, 13-17)* and further genes (*GAL2, MAL11, MPH2,3) [*[*7*](#_ENREF_7)*]* | *YFL011W*  *YOL156W*  *YEL069C*  *YNL318C*  *YDL245C*  *YJR158W*  *YNR072W*  *YDR345C*  *YHR092C*  *YHR096C*  *YDR343C*  *YDR342C*  *YJL214W*  *YJL219W*  *YHR094C*  *YMR011W*  *YLR081W*  *YGR289C*  *YDL247W*  *YJR160C* | *HXT10*  *HXT11*  *HXT13*  *HXT14*  *HXT15*  *HXT16*  *HXT17*  *HXT3*  *HXT4*  *HXT5*  *HXT6*  *HXT7*  *HXT8*  *HXT9*  *HXT1*  *HXT2*  *GAL2*  *MAL11*  *MPH2*  *MPH3* | ==> glucose |
| Transport | *-* | - | ethanol[c] ==> |
| Transport | *-* | - | glycerol ==> |
| Transport | *-* | - | acetate[c] ==> |
| Transport | *-* | - | CO2 <==> |
| Transport | *-* | - | ==> O2 |
| Artificial IPP production | *-* | - | isopentenyl-diphosphate ==> |
| Growth in mmol*gCDW^-1^*h^-1^ | *-* | - | 0.1 BIOMASS ==> |
| **Glycolysis** | | | |
| Glucose kinase [EC 2.7.1.2], hexokinase [EC:2.7.1.1] | *YCL040W*  *YFR053C*  *YGL253W* | *GLK1*  *HXK1*  *HXK2* | ATP + glucose ==> ADP + glucose-6-P |
| Glucose-6-phosphate isomerase [EC:5.3.1.9] | *YBR196C* | *PGI1* | glucose-6-P <==> fructose-6-P |
| 6-phosphofructokinase [EC:2.7.1.11] | *YGR240C*  *YMR205C* | *PFK1*  *PFK2* | ATP + fructose-6-P ==> ADP + fructose-1,6-bis-P |
| Fructose-bisphosphate aldolase, class II [EC:4.1.2.13] | *YKL060C* | *FBA1* | fructose-1,6-bis-P <==> DHAP + GA-3-P |
| Triosephosphate isomerase (TIM) [EC:5.3.1.1] | *YDR050C* | *TPI1* | DHAP <==> GA-3-P |
| Glyceraldehyde 3-phosphate dehydrogenase [EC:1.2.1.12] | *YJL052W*  *YJR009C*  *YGR192C* | *TDH1*  *TDH2*  *TDH3* | GA-3-P + NAD[c] <==> 1,3-P-glycerate + NADH[c] |
| Phosphoglycerate kinase [EC:2.7.2.3] | *YCR012W* | *PGK1* | 1,3-P-glycerate + ADP <==> 3-P-glycerate + ATP |
| Phosphoglycerate mutase [EC:5.4.2.1] | *YKL152C*  *YDL021W*  *YOL056W* | *GPM1*  *GPM2*  *GPM3* | 3-P-glycerate <==> 2-P-glycerate |
| Enolase [EC:4.2.1.11] | *YGR254W*  *YHR174W* | *ENO1*  *ENO2* | 2-P-glycerate <==> PEP |
| Pyruvate kinase [EC:2.7.1.40] | *YAL038W*  *YOR347C* | *PYK1*  *PYK2* | ADP + PEP ==> ATP + pyruvate[c] |
| **Pentose phosphate pathway** | | | |
| Glucose-6-phosphate 1-dehydrogenase [EC:1.1.1.49] | *YNL241C* | *ZWF1* | NADP[c] + glucose-6-P ==> G15L + NADPH[c] |
| 6-phosphogluconolactonase [EC:3.1.1.31] | *YNR034W*  *YCR073W-A*  *YHR163W*  *YGR248W* | *SOL1*  *-*  *SOL3*  *SOL4* | G15L ==> gluconate-6-P |
| 6-phosphogluconate dehydrogenase [EC:1.1.1.44] | *YGR256W*  *YHR183W* | *GND2*  *GND1* | NADP[c] + gluconate-6-P ==> CO2 + NADPH[c] + ribulose-5-P |
| Ribose 5-phosphate isomerase A [EC:5.3.1.6] | *YOR095C* | *RKI1* | ribulose-5-P <==> ribose-5-P |
| Ribulose-phosphate 3-epimerase [EC:5.1.3.1] | *YJL121C* | *RPE1* | ribulose-5-P <==> xylulose-5-P |
| Transketolase [EC:2.2.1.1] | *YBR117C*  *YPR074C* | *TKL2*  *TKL1* | ribose-5-P + xylulose-5-P <==> GA-3-P + sedoheptulose-7-P |
| Transaldolase [EC:2.2.1.2] | *YGR043C* | *NOM1* | GA-3-P + sedoheptulose-7-P <==> erythrose-4-P + fructose-6-P |
| Transketolase [EC:2.2.1.1] | *YBR117C*  *YPR074C* | *TKL2*  *TKL1* | erythrose-4-P + xylulose-5-P <==> GA-3-P + fructose-6-P |
| **Gluconeogenesis, anaplerotic reactions, pyruvate dehydrogenase bypass and side product formation** | | | |
| Fructose-1,6-bisphosphatase [EC:3.1.3.11] | *YLR377C* | *FBP1* | fructose-1,6-bis-P ==> fructose-6-P |
| Pyruvate carboxylase [EC:6.4.1.1] | *YGL062W*  *YBR218C* | *PYC1*  *PYC2* | ATP + CO2 + pyruvate[c] ==> ADP + oxaloacetate[c] |
| Phosphoenolpyruvate carboxykinase [EC:4.1.1.49] | *YKR097W* | *PCK1* | ATP + oxaloacetate[c] ==> ADP + CO2 + PEP |
| Pyruvate decarboxylase [EC:[4.1.1.1](http://www.genome.jp/dbget-bin/www_bget?ec:4.1.1.1)] | *YLR044C*  *YLR134W*  *YGR087C* | *PDC1*  *PDC5*  *PDC6* | pyruvate[c] ==> CO2 + acetaldehyde[c] |
| Alcohol dehydrogenase [EC:[1.1.1.1](http://www.genome.jp/dbget-bin/www_bget?ec:1.1.1.1)] | *YOL086C*  *YMR303C*  *YGL256W*  *YBR145W* | *ADH1*  *ADH2*  *ADH4*  *ADH5* | NADH[c] + acetaldehyde[c] <==> NAD[c] + ethanol[c] |
| Mg(2+)-ACDH; Mg(2+)-activated acetaldehyde dehydrogenase [EC:[1.2.1.3](http://www.genome.jp/dbget-bin/www_bget?ec:1.2.1.3)] | *YPL061W* | *ALD6* | NADP[c] + acetaldehyde[c] ==> NADPH[c] + acetate[c] |
| Aldehyde dehydrogenase 1 and 2 [EC:1.2.1.5] | *YMR170C*  *YMR169C* | *ALD2*  *ALD3* | NAD[c] + acetaldehyde[c] ==> NADH[c] + acetate[c] |
| Acetyl-CoA synthetase [EC:[6.2.1.1](http://www.genome.jp/dbget-bin/www_bget?ec:6.2.1.1)] | *YLR153C* | *ACS2* | ATP + acetate[c] ==> AMP + AcCoA[c] |
| Glycerol-3-phosphate dehydrogenase [EC 1.1.1.8] | *YDL022W*  *YOL059W* | *GPD1*  *GPD2* | DHAP + NADH[c] <==> NAD[c] + glycerol-P |
| Glycerol-3-phosphatase 2 [EC:3.1.3.21] | *YER062C*  *YIL053W* | *HOR2*  *RHR2* | glycerol-P ==> glycerol |
| **Pyruvate dehydrogenase complex, citric acid cycle and mitochondrial reactions** | | | |
| Pyruvate dehydrogenase complex:E2 (Lat1p) core [EC:2.3.1.12], E1 (Pda1p and Pdb1p) [EC:1.2.4.1], E3 (Lpd1p) and Protein X (Pdx1p) | *YBR221C*  *YNL071W*  *YGR193C*  *YER178W*  *YFL018C* | *PDB1*  *LAT1*  *PDX1*  *PDA1*  *LPD1* | NAD[m] + pyruvate[m] ==> AcCoA[m] + CO2 + NADH[m] |
| Citrate synthase [EC:2.3.3.1] | *YNR001C*  *YPR001W* | *CIT1*  *CIT3* | AcCoA[m] + oxaloacetate[m] ==> citrate |
| Aconitate hydratase 1 [EC:4.2.1.3] | *YLR304C* | *ACO1* | citrate <==> isocitrate |
| Isocitrate dehydrogenase [NAD] [EC:1.1.1.41] | *YNL037C*  *YOR136W* | *IDH1*  *IDH2* | NAD[m] + isocitrate ==> CO2 + NADH[m] + a-ketoglutarate |
| Isocitrate dehydrogenase [NADP] [EC:1.1.1.42) | *YDL066W* | *IDP1* | NADP[m] + isocitrate ==> CO2 + NADPH[m] + a-ketoglutarate |
| Alpha-ketoglutarate dehydrogenase, Dihydrolipoyl dehydrogenase [EC:1.2.4.2], [EC:1.8.1.4] | *YIL125W*  *YDR148C*  *YFL018C* | *KGD1*  *KGD2*  *LPD1* | NAD[m] + a-ketoglutarate ==> CO2 + NADH[m] + succinyl-CoA |
| Succinyl-CoA ligase [EC:6.2.1.5] | *YOR142W*  *YGR244C* | *LSC1*  *LSC2* | ADP + succinyl-CoA <==> ATP + succinate |
| Succinate dehydrogenase complex [EC:1.3.5.1] | *YDR178W*  *YKL141W*  *YKL148C*  *YLL041C*  *YJL045W* | *SDH4*  *SDH3*  *SDH1*  *SDH2*  *-* | FAD + succinate <==> FADH2 + fumarate |
| Fumarate hydratase [EC:4.2.1.2] | *YPL262W* | *FUM1* | fumarate <==> malate |
| Malate dehydrogenase [EC:1.1.1.37] | *YKL085W* | *MDH1* | NAD[m] + malate <==> NADH[m] + oxaloacetate[m] |
| Malic enzyme [EC:1.1.1.38] | *YKL029C* | *MAE1* | NADP[m] + malate ==> CO2 + NADPH[m] + pyruvate[m] |
| Malic enzyme [EC:1.1.1.38] | *YKL029C* | *MAE1* | NAD[m] + malate ==> CO2 + NADH[m] + pyruvate[m] |
| Alcohol dehydrogenases *ADH1-5* [EC:1.1.1.1] | *YOL086C*  *YMR303C*  *YMR083W*  *YGL256W*  *YBR145W* | *ADH1*  *ADH2*  *ADH3*  *ADH4*  *ADH5* | NAD[m] + ethanol[m] <==> NADH[m] + acetaldehyde[m] |
| Potassium-activated aldehyde dehydrogenase [EC:1.2.1.3] | *YOR374W* | *ALD4* | NAD[m] + acetaldehyde[m] ==> NADH[m] + acetate[m] |
| Potassium-activated aldehyde dehydrogenase, Aldehyde dehydrogenase 5 [EC:1.2.1.3] | *YOR374W*  *YER073W* | *ALD4*  *ALD5* | NADP[m] + acetaldehyde[m] ==> NADPH[m] + acetate[m] |
| Acetyl-CoA synthetase 1-2 [EC:6.2.1.1] | *YAL054C* | *ACS1* | ATP + acetate[m] ==> AMP + AcCoA[m] |
| **Mitochondrial shuttles** | | | |
| NAD/NADH shuttle [[8](#_ENREF_8)] | *-* |  | NADH[c] + NAD[m] ==> NADH[m] + NAD[c] |
| Transport: mitochondrial pyruvate carrier | *YGL080W* | *FMP37* | pyruvate[c] <==> pyruvate[m] |
| Transport: mitochondrial inner membrane transporter | *YKL120W* | *OAC1* | oxaloacetate[c] <==> oxaloacetate[m] |
| Transport | *-* |  | ethanol[c] <==> ethanol[m] |
| Transport | *-* |  | acetaldehyde[c] <==> acetaldehyde[m] |
| Transport | *-* |  | acetate[c] <==> acetate[m] |
| **MVA pathway** | | | |
| Acetyl-CoA C-acetyltransferase [EC:2.3.1.9] | *YPL028W* | *ERG10* | 2 AcCoA[c] <==> acetoacetyl-CoA |
| Hydroxymethylglutaryl-CoA synthase [EC:2.3.3.10] | *YML126C* | *ERG13* | AcCoA[c] + acetoacetyl-CoA <==> 3-hydroxy-3-methylglutaryl-CoA |
| Hydroxymethylglutaryl-CoA reductase (NADPH) [EC:1.1.1.34] | *YML075C*  *YLR450W* | *HMG1*  *HMG2* | 3-hydroxy-3-methylglutaryl-CoA + 2 NADPH[c] <==> 2 NADP[c] + mevalonate |
| Mevalonate kinase [EC:2.7.1.36] | *YMR208W* | *ERG12* | ATP + mevalonate ==> 5-phosphomevalonate + ADP |
| Phosphomevalonate kinase [EC:2.7.4.2] | *YMR220W* | *ERG8* | 5-phosphomevalonate + ATP ==> 5-diphosphomevalonate + ADP |
| Diphosphomevalonate decarboxylase [EC:4.1.1.33] | *YNR043W* | *ERG19* | 5-diphosphomevalonate + ATP <==> ADP + CO2 + isopentenyl-diphosphate |
| **Oxidative phosphorylation and ATP maintenance** | | | |
| Respiratory chain: NADH dehydrogenase [EC:1.6.5.3] and F1F0 ATP synthase [EC:3.6.3.14] | *YMR145C*  *YDL085W*  *YBL099W*  *YDL004W*  *YDR377W*  *YJR121W*  *YKL016C*  *YLR295C*  *Q0085*  *Q0130*  *YPL271W* | *NDE1*  *NDE2*  *ATP1*  *ATP16*  *ATP17*  *ATP2*  *ATP7*  *ATP14*  *ATP6*  *ATP9*  *ATP15* | 24 ADP + 20 NADH[m] + 10 O2 ==> 24 ATP + 20 NAD[m] |
| Respiratory chain: Succinate dehydrogenase complex [EC:1.3.5.1] and F1F0 ATP synthase [EC:3.6.3.14] | *YDR178W*  *YKL141W*  *YKL148C*  *YLL041C YJL045W*  *YBL099W*  *YDL004W*  *YDR377W*  *YJR121W*  *YKL016C*  *YLR295C*  *Q0085*  *Q0130*  *YPL271W* | *SDH4*  *SDH3*  *SDH1*  *SDH2*  *-*  *ATP1*  *ATP16*  *ATP17*  *ATP2*  *ATP7*  *ATP14*  *ATP6*  *ATP9*  *ATP15* | 24 ADP + 20 FADH2 + 10 O2 ==> 24 ATP + 20 FAD |
| ATP maintenace | *-* | *-* | ATP ==> ADP |
| Adenylate kinase [EC:2.7.4.3] | *YER170W*  *YDR226W* | *ADK2*  *ADK1* | AMP + ATP ==> 2 ADP |
| **Biomass formation** | | | |
| Biomass formation [[8](#_ENREF_8), [9](#_ENREF_9)] | *-* | - | 6 3-P-glycerate + 254 ATP + 24 AcCoA[c] + 3 AcCoA[m] + 90 NADPH[c] + 22 NADPH[m] + 16 NAD[c] + 6 NAD[m] + 6 PEP + 11 a-ketoglutarate + 3 erythrose-4-P + 25 glucose-6-P + glycerol-P + 10 oxaloacetate[c] + 18 pyruvate[c] + 3 ribose-5-P ==> 254 ADP + BIOMASS + 16 NADH[c] + 6 NADH[m] + 90 NADP[c] + 22 NADP[m] |

**Artificial sources**

| **Enzyme/Description** | **Gene** | **Reaction** |
| --- | --- | --- |
| Artificial ATP source | - | ADP ==> ATP |
| Artificial NADPH source | - | NADP[c] ==> NADPH[c] |
| Artificial NADH source | - | NAD[c] ==> NADH[c] |

**Heterologous enzymes/pathways**

| **Enzyme/Description** | **Gene** | **Reaction** |
| --- | --- | --- |
| Heterologous soluble transhydrogenase from *E. coli* | *sthA/udhA* | NADH[c] + NADP[c] <==> NADPH[c] + NAD[c] |
| Cytosolic pyruvate dehydrogenase complex from *E. coli* | *lpdA*  *aceEF* | NAD[c] + pyruvate[c] ==> AcCoA[c] + CO2 + NADH[c] |
| NADP+-dependent glyceraldehyde-3-phosphate dehydrogenase [EC:1.2.1.13]; e.g. from *K. lactis* [[10](#_ENREF_10)] | *GDP1* | GA-3-P + NADP[c] <==> 1,3-P-glycerate + NADPH[c] |
| Transfer of the native MVA pathway into mitochondria | *YPL028W / ERG10* | 2 AcCoA[m] <==> acetoacetyl-CoA |
|  | *YML126C / ERG13* | AcCoA[m] + acetoacetyl-CoA <==> 3-hydroxy-3-methylglutaryl-CoA |
|  | *YML075C / HMG1*  *YLR450W / HMG2* | 3-hydroxy-3-methylglutaryl-CoA + 2 NADPH[m] <==> 2 NADP[m] + mevalonate |
|  | *YMR208W / ERG12* | ATP + mevalonate ==> 5-phosphomevalonate + ADP |
|  | *YMR220W / ERG8* | 5-phosphomevalonate + ATP ==> 5-diphosphomevalonate + ADP |
|  | *YNR043W / ERG19* | 5-diphosphomevalonate + ATP <==> ADP + CO2 + isopentenyl-diphosphate |
| Heterologous ATP-citrate-lyase [EC:2.3.3.8], e.g. from *Yarrowia lipolytica* | *ACL1* | Cit + ATP ==> oxaloacetate[c] + AcCoA[c] + ADP |
| Introduction of the DXP pathway from *E. coli* | *dxs, dxr/ispC, ispDEFGH* | 2 ATP + GA-3-P + 3 NADPH[c] + pyruvate[c] ==> 2 ADP + CO2 + 3 NADP[c] + DMAPP |
|  | *idi* | DMAPP <==> isopentenyl-diphosphate |

**Different carbon sources**

| **Enzyme/Description** | **Gene (systematic name)** | **Gene (standard name)** | **Reaction** |
| --- | --- | --- | --- |
| **Xylose** | | | |
| Transport by *HXT* genes or heterologous transporters [[11](#_ENREF_11)] | *-* | *-* | ==> Xylose |
| xylulose kinase [EC:2.7.1.17] | *YGR194C* | *XKS1* | ATP + Xylulose ==> ADP + xylulose-5-P |
| XI pathway: xylose isomerase [EC:5.3.1.5], e.g. from *Piromyces sp.* [[12](#_ENREF_12)] | *-* | *xylA* | Xylose ==> Xylulose |
| XR-XDH pathway: xylose reductase (XR) [EC:1.1.1.307] and xylitol dehydrogenase (XDH) [EC:1.1.1.9], e.g. from *Pichia stipites* [[12](#_ENREF_12)] | *-* | *XYL1* | NADH[c] + Xylose ==> NAD[c] + xylitol |
|  | *-* | *XYL1* | NADPH[c] + Xylose ==> NADP[c] + xylitol |
|  | *-* | *XYL2* | NAD[c] + xylitol ==> NADH[c] + Xylulose |
| **Galactose** | | | |
| Transport: see glucose | **-** | *-* | ==> galactose |
| Galactokinase [EC:2.7.1.6], galactose-1-P-uridylyltransferase [EC:2.7.7.12], UDP-glucose-4-epimerase [EC:5.1.3.2], phosphoglucomutase-1 & 2 [EC:5.4.2.2] | *YBR020W*  *YDR009W*  *YBR018C*  *YBR019C*  *YKL127W*  *YMR105C*  *YMR278W* | *GAL1*  *GAL3*  *GAL7*  *GAL10*  *PGM1*  *PGM2*  *PRM15* | galactose + ATP ==> glucose-6-P + ADP |
| **Fructose** | | | |
| Transport: see glucose | *-* | *-* | ==> fructose |
| Hexokinase [EC:2.7.1.1] | *YGL253W* | *HXK2* | fructose + ATP ==> ADP + fructose-6-P |
| **Ethanol** |  |  |  |
| Transport by diffusion [[13](#_ENREF_13)] | *-* | *-* | ==> ethanol[c] |
| **Glycerol** |  |  |  |
| Transport | *-* | *-* | ==> glycerol |
| glycerol kinase [EC:2.7.1.30] | *YHL032C* | *GUT1* | glycerol + ATP ==> glycerol-P + ADP |
| **glyoxylate cycle plus mitochondrial shuttles plus compartmentalization of intermediates of citric acid cycle for growth on ethanol and glycerol** | | | |
| Citrate synthase [EC:2.3.3.1] | *YCR005C* | *CIT2* | AcCoA[c] + oxaloacetate[c] ==> citrate[c] |
| Aconitate hydratase 1 [EC:4.2.1.3] | *YLR304C* | *ACO1* | citrate[c] <==> isocitrate[c] |
| Isocitrate lyase [EC:4.1.3.1] | *YER065C*  *YPR006C* | *ICL1*  *ICL2* | isocitrate[c] ==> succinate[c] + glyoxylate |
| Malate synthase 1 [EC:2.3.3.9] | *YNL117W*  *YIR031C* | *MLS1*  *MLS2* | AcCoA[c] + glyoxylate ==> malate[c] |
| Malate dehydrogenase [EC:1.1.1.37] | *YOL126C*  *YDL078C* | *MDH2*  *MDH3* | NAD[c] + malate[c] <==> NADH[c] + oxaloacetate[c] |
| Fumarate hydratase [EC:4.2.1.2] | *YPL262W* | *FUM1* | malate[c] <==> fumarate[c] |
| Fumarate reductase [EC:1.3.1.6] | *YJR051W*  *YEL047C* | *OSM1*  *FRD1* | NADH[c] + fumarate[c] ==> NAD[c] + succinate[c] |
| Isocitrate dehydrogenase [EC:1.1.1.42] | *YNL009W*  *YLR174W* | *IDP3*  *IDP2* | NADP[c] + isocitrate[c] ==> CO2 + NADPH[c] + a-ketoglutarate[c] |
| Transport: mitochondrial dicarboxylate transporter | *YLR348C* | *DIC1* | succinate[c] ==> succinate[m] |
| Transport: mitochondrial citrate-oxoglutarate carrier | *YMR241W* | *YHM2* | a-ketoglutarate[c] + citrate[m] ==> citrate[c] + a-ketoglutarate[m] |
| Transport: mitochondrial succinate-fumarate transporte | *YJR095W* | *SFC1* | succinate[c] + fumarate[m] ==> fumarate[c] + succinate[m] |
| Transport: mitochondrial inner membrane citrate transporter | *YBR291C* | *CTP1* | citrate[c] + malate[m] <==> malate[c] + citrate[m] |
| Transport: mitochondrial inner membrane citrate transporter | *YBR291C* | *CTP1* | citrate[c] + isocitrate[m] <==> isocitrate[c] + citrate[m] |
| Transport: mitochondrial dicarboxylate transporter | *YLR348C* | *DIC1* | malate[c] <==> malate[m] |
| Transport: carnitine O-acetyltransferase [EC:2.3.1.7] | *YOR100C*  *YAR035W*  *YER024W*  *YML042W* | *CRC1*  *YAT1*  *YAT2*  *CAT2* | AcCoA[c] ==> AcCoA[m] |
| Citrate synthase [EC:2.3.3.1] | *YNR001C*  *YPR001W* | *CIT1*  *CIT3* | AcCoA[m] + oxaloacetate[m] ==> citrate[m] |
| Aconitate hydratase 1 [EC:4.2.1.3] | *YLR304C* | *ACO1* | citrate[m] <==> isocitrate[m] |
| Isocitrate dehydrogenase [NAD] [EC:1.1.1.41] | *YNL037C*  *YOR136W* | *IDH1*  *IDH2* | NAD[m] + isocitrate[m] ==> CO2 + NADH[m] + a-ketoglutarate[m] |
| Isocitrate dehydrogenase [NADP] [EC:1.1.1.42) | *YDL066W* | *IDP1* | NADP[m] + isocitrate[m] ==> CO2 + NADPH[m] + a-ketoglutarate[m] |
| Alpha-ketoglutarate dehydrogenase, Dihydrolipoyl dehydrogenase [EC:1.2.4.2], [EC:1.8.1.4] | *YIL125W*  *YDR148C*  *YFL018C* | *KGD1*  *KGD2*  *LPD1* | NAD[m] + a-ketoglutarate[m] ==> CO2 + NADH[m] + succinyl-CoA |
| Succinyl-CoA ligase [EC:6.2.1.5] | *YOR142W*  *YGR244C* | *LSC1*  *LSC2* | ADP + succinyl-CoA <==> ATP + succinate[m] |
| Succinate dehydrogenase [EC:1.2.4.2], [EC:1.8.1.4], [EC:2.3.1.61] | *YDR178W*  *YKL141W*  *YJL045W*  *YKL148C*  *YLL041C* | *SDH4*  *SDH3*  *SDH1*  *SDH2*  *-* | FAD + succinate[m] <==> FADH2 + fumarate[m] |
| Fumarate hydratase [EC:4.2.1.2] | *YPL262W* | *FUM1* | fumarate[m] <==> malate[m] |
| Malate dehydrogenase [EC:1.1.1.37] | *YKL085W* | *MDH1* | NAD[m] + malate[m] <==> NADH[m] + oxaloacetate[m] |
| Malic enzyme [EC:1.1.1.38] | *YKL029C* | *MAE1* | NADP[m] + malate[m] ==> CO2 + NADPH[m] + pyruvate[m] |
| Malic enzyme [EC:1.1.1.38] | *YKL029C* | *MAE1* | NAD[m] + malate[m] ==> CO2 + NADH[m] + pyruvate[m] |

***E. coli***

**Wild type network on glucose**

| **Enzyme/Description** | **Gene** | **Reaction** |
| --- | --- | --- |
| **Transport** | | |
| Transport | - | ==> glucose |
| Glucose uptake by phosphoenolpyruvate:glucose transferase system | *ptsGHI, crr* | PEP + glucose ==> glucose-6-P + pyruvate |
| Transport | - | ==> O2 |
| Transport | - | ==> NH3 |
| Transport | - | ethanol ==> |
| Transport | - | lactate ==> |
| Transport | - | succinate ==> |
| Transport | - | acetate ==> |
| Transport | - | formate ==> |
| Transport | - | CO2 <==> |
| Transport | - | H2 ==> |
| Growth in mmol*gCDW^-1^*h^-1^ | - | 0.001 BIOMASS ==> |
| Artificial IPP production | - | isopentenyl-diphosphate ==> |
| **Glycolysis** | | |
| Glucose-6-phosphate isomerase [EC:5.3.1.9] | *pgi* | glucose-6-P <==> fructose-6-P |
| 6-phosphofructokinase [EC:2.7.1.11] | *pfkAB* | ATP + fructose-6-P ==> ADP + fructose-1,6-bis-P |
| Fructose-bisphosphate aldolase, class I [EC:4.1.2.13] | *fbaAB* | fructose-1,6-bis-P <==> DHAP + GA-3-P |
| Triosephosphate isomerase [EC:5.3.1.1] | *tpiA* | DHAP <==> GA-3-P |
| Glyceraldehyde 3-phosphate dehydrogenase [EC:1.2.1.12] | *gapA* | GA-3-P + NAD <==> 1,3-P-glycerate + NADH |
| Phosphoglycerate kinase [EC:2.7.2.3] | *pgk* | 1,3-P-glycerate + ADP <==> 3-P-glycerate + ATP |
| Phosphoglycerate mutase [EC:5.4.2.1] | *gpmM, ytjC, gpmA* | 3-P-glycerate <==> 2-P-glycerate |
| Enolase [EC:4.2.1.11] | *eno* | 2-P-glycerate <==> PEP |
| Pyruvate kinase [EC:2.7.1.40] | *pykAF* | ADP + PEP ==> ATP + pyruvate |
| **Pentose phosphate pathway** | | |
| Glucose-6-phosphate 1-dehydrogenase [EC:1.1.1.49] | *zwf* | NADP + glucose-6-P ==> G15L + NADPH |
| 6-phosphogluconolactonase [EC:3.1.1.31] | *pgl* | G15L ==> gluconate-6-P |
| 6-phosphogluconate dehydrogenase [EC:1.1.1.44] | *gnd* | NADP + gluconate-6-P ==> CO2 + NADPH + ribulose-5-P |
| Ribose 5-phosphate isomerase A [EC:5.3.1.6] | *rpiAB* | ribulose-5-P <==> ribose-5-P |
| Ribulose-phosphate 3-epimerase [EC:5.1.3.1] | *rpe* | ribulose-5-P <==> xylulose-5-P |
| Transketolase [EC:2.2.1.1] | *tktAB* | ribose-5-P + xylulose-5-P <==> GA-3-P + sedoheptulose-7-P |
| Transaldolase [EC:2.2.1.2] | *talAB* | GA-3-P + sedoheptulose-7-P <==> erythrose-4-P + fructose-6-P |
| Transketolase [EC:2.2.1.1] | *tktAB* | erythrose-4-P + xylulose-5-P <==> GA-3-P + fructose-6-P |
| **Entner-Doudoroff/KDPG pathway** | | |
| Phosphogluconate dehydratase [EC:4.2.1.12] | *edd* | gluconate-6-P ==> 2-keto-3-deoxy-6-phospho-gluconate |
| 2-keto-3-deoxy-6-phosphogluconate aldolase [EC:4.1.3.16] | *eda* | 2-keto-3-deoxy-6-phospho-gluconate ==> GA-3-P + pyruvate |
| **Gluconeogenesis** | | |
| Fructose-1,6-biphosphatase [EC:3.1.3.11] | *glpX, fbp* | fructose-1,6-bis-P ==> fructose-6-P |
| Phosphoenolpyruvate synthase [EC:2.7.9.2] | *pps* | ATP + pyruvate ==> AMP + PEP |
| **Pyruvate dehydrogenase complex and citric acid cycle** | | |
| Pyruvate dehydrogenase complex [EC:1.2.4.1] [EC:2.3.1.12] [EC:1.8.1.4] | *lpdA, aceEF* | NAD + pyruvate ==> AcCoA + CO2 + NADH |
| Citrate synthase [EC:2.3.3.1] | *prpC, gltA* | AcCoA + oxaloacetate ==> citrate |
| Aconitate hydratase 1 [EC:4.2.1.3] | *acnAB* | citrate <==> isocitrate |
| Isocitrate dehydrogenase [NADP] [EC:1.1.1.42] | *icd* | NADP + isocitrate ==> CO2 + NADPH + a-ketoglutarate |
| Alpha-ketoglutarate dehydrogenase complex [EC:1.2.4.2] [EC:1.8.1.4] [EC:2.3.1.61] | *lpdA, sucAB* | NAD + a-ketoglutarate ==> CO2 + NADH + succinyl-CoA |
| Succinyl-CoA synthetase [EC:6.2.1.5] | *sucCD* | ADP + succinyl-CoA <==> ATP + succinate |
| Succinate dehydrogenase [EC:1.3.5.1] and fumarate reductase [EC:1.3.5.4] | *sdhABCD*  *frdABCD* | succinate + quinine <==> fumarate + quinol |
| Fumarate hydratase [EC:4.2.1.2] | *fumABC* | fumarate <==> malate |
| Malate dehydrogenase [EC:1.1.1.37] | *mdh* | NAD + malate <==> NADH + oxaloacetate |
| Malate:quinone oxidoreductase [EC:1.1.99.16] | *mqo* | malate + quinine ==> oxaloacetate + quinol |
| **Anaplerotic reactions** | | |
| Malic enzyme [EC 1.1.1.38] | *maeA* | NAD + malate ==> CO2 + NADH + pyruvate |
| Malic enzyme [EC 1.1.1.40] | *maeB* | NADP + malate ==> CO2 + NADPH + pyruvate |
| Phosphoenolpyruvate carboxylase [EC:4.1.1.31] | *ppc* | CO2 + PEP ==> oxaloacetate |
| Phosphoenolpyruvate carboxykinase [EC:4.1.1.49] | *pck* | ATP + oxaloacetate ==> ADP + CO2 + PEP |
| **Mixed acid fermentation** | | |
| D-lactate dehydrogenase [EC:1.1.1.28] | *ldhA* | NADH + pyruvate ==> NAD + lactate |
| Acetaldehyde dehydrogenase/alcohol dehydrogenase [EC:1.2.1.10] [EC:1.1.1.1] | *adhE* | AcCoA + NADH ==> NAD + acetaldehyde |
| Alcohol dehydrogenase [EC:1.1.1.1] | *adhE*  *adhP* | NADH + acetaldehyde ==> NAD + ethanol |
| Phosphate acetyltransferase [EC:2.3.1.8] | *pta* | AcCoA ==> acetylphosphate |
| Acetate kinase [EC:2.7.2.1] | *ackAB* | ADP + acetylphosphate ==> ATP + acetate |
| pyruvate dehydrogenase (pyruvate oxidase) [EC:1.2.2.2] | *poxB* | pyruvate + quinine ==> CO2 + acetate + quinol |
| pyruvate formate-lyase [EC:2.3.1.54] | *pflB, tdcE* | pyruvate ==> AcCoA + formate |
| Formate hydrogenylase complex [EC:1.12.99.6] [EC:1.1.99.33] | *hycBCDEFG, fdhF* | formate ==> CO2 + H2 |
| **DXP pathway** | | |
| 1-deoxyxylulose-5-phosphate synthase [EC:[2.2.1.7](http://www.genome.jp/dbget-bin/www_bget?ec:2.2.1.7)]  1-deoxy-D-xylulose 5-phosphate reductoisomerase (uses NADPH [[14](#_ENREF_14)]) [EC:1.1.1.267]  4-diphosphocytidyl-2C-methyl-D-erythritol synthase (uses CTP) [EC:2.7.7.60]  4-diphosphocytidyl-2-C-methylerythritol kinase (uses ATP) [EC:[2.7.1.148](http://www.genome.jp/dbget-bin/www_bget?ec:2.7.1.148)]  2C-methyl-D-erythritol 2,4-cyclodiphosphate synthase [EC:[4.6.1.12](http://www.genome.jp/dbget-bin/www_bget?ec:4.6.1.12)]  1-hydroxy-2-methyl-2-(E)-butenyl 4-diphosphate synthase (uses NADPH [[15](#_ENREF_15)]) [EC:[1.17.7.1](http://www.genome.jp/dbget-bin/www_bget?ec:1.17.7.1)]  1-hydroxy-2-methyl-2-(E)-butenyl 4-diphosphate reductase (uses NADPH [[16](#_ENREF_16), [17](#_ENREF_17)]) [EC:[1.17.1.2](http://www.genome.jp/dbget-bin/www_bget?ec:1.17.1.2)] | *dxs, dxr/ispC, ispDEFGH* | 2 ATP + GA-3-P + 3 NADPH + pyruvate ==> 2 ADP + CO2 + 3 NADP + dimethylallyl-diphosphate |
| isopentenyl-diphosphate delta-isomerase [EC:5.3.3.2] | *idi* | dimethylallyl-diphosphate <==> isopentenyl-diphosphate |
| **Oxidative phosphorylation and ATP maintenance** | | |
| Respiratory chain: NADH dehydrogenase I [EC:1.6.5.3] and ATP synthase [EC:3.6.3.14] | *nuoAHJKLMNEFGBCI, atpABCDEFGHI* | 2 ADP + NADH + O2 ==> 2 ATP + NAD |
| Respiratory chain: NADH dehydrogenase II [EC:1.6.5.8] | *ndh* | NADH + quinine <==> NAD + quinol |
| Respiratory chain: Cytochrome *bo* terminal oxidase [EC:1.10.3.10] and ATP synthase [EC:3.6.3.14] | *cyoABCD, atpABCDEFGHI* | ADP + O2 + quinol ==> ATP + quinine |
| Pyridine nucleotide transhydrogenase, membrane-bound [EC:1.6.1.2] (*sthA/udhA* [EC:1.6.1.1] is ignored) | *pntAB* | ADP + 3 NADPH + 3 NAD <==> ATP + 3 NADH + 3 NADP |
| Adenylate kinase [EC:2.7.4.3] | *adk* | AMP + ATP ==> 2 ADP |
| ATP maintenance | *-* | ATP ==> ADP |
| **Biomass formation** | | |
| Biomass formation [[18](#_ENREF_18), [19](#_ENREF_19)] | - | 1642 3-P-glycerate + 40680 ATP + 1207 AcCoA + 31 GA-3-P + 18320 NADPH + 4079 NAD + 960 PEP + 1427 a-ketoglutarate + 512 erythrose-4-P + 17 fructose-6-P + 49 glucose-6-P + 2355 oxaloacetate + 3920 pyruvate + 860 ribose-5-P + 12502 NH3 ==> 40680 ADP + BIOMASS + 4079 NADH + 18320 NADP |

**Artificial sources**

| **Enzyme/Description** | **Gene** | **Reaction** |
| --- | --- | --- |
| Artificial ATP source | - | ADP ==> ATP |
| Artificial NADPH source | - | NADP[c] ==> NADPH[c] |
| Artificial NADH source | - | NAD[c] ==> NADH[c] |

**Heterologous enzymes/pathways**

| **Enzyme/Description** | **Gene** | **Reaction** |
| --- | --- | --- |
| NADP+-dependent glyceraldehyde-3-phosphate dehydrogenase [EC:1.2.1.13]; e.g. from *K. lactis [*[*10*](#_ENREF_10)*]* | *GDP1* | GA-3-P + NADP <==> 1,3-P-glycerate + NADPH |
| MVA pathway from yeast | *YPL028W / ERG10* | 2 AcCoA <==> acetoacetyl-CoA |
|  | *YML126C / ERG13* | AcCoA + acetoacetyl-CoA <==> 3-hydroxy-3-methylglutaryl-CoA |
|  | *YML075C / HMG1*  *YLR450W / HMG2* | 3-hydroxy-3-methylglutaryl-CoA + 2 NADPH <==> 2 NADP + mevalonate |
|  | *YMR208W / ERG12* | ATP + mevalonate ==> 5-phosphomevalonate + ADP |
|  | *YMR220W / ERG8* | 5-phosphomevalonate + ATP ==> 5-diphosphomevalonate + ADP |
|  | *YNR043W / ERG19* | 5-diphosphomevalonate + ATP <==> ADP + CO2 + isopentenyl-diphosphate |

**Different carbon source**

| **Enzyme/Description** | **Gene** | **Reaction** |
| --- | --- | --- |
| **Glycerol plus glyoxylate shunt** | | |
| Transport | *-* | ==> glycerol |
| Glycerol uptake by glycerol kinase [EC:2.7.1.30] | *glpK* | ATP + glycerol ==> ADP + glycerol-P |
| Glycerol-3-phosphate dehydrogenase [EC:1.1.1.94] | *gpsA* | NADP + glycerol-P <==> DHAP + NADPH |
| Isocitrate lyase [EC:4.1.3.1] | *aceA* | isocitrate ==> succinate + glyoxylate |
| Malate synthase [EC:2.3.3.9] | *aceB, glcB* | AcCoA + glyoxylate ==> malate |

**References**

1. Keseler IM, Collado-Vides J, Santos-Zavaleta A, Peralta-Gil M, Gama-Castro S, Muniz-Rascado L, Bonavides-Martinez C, Paley S, Krummenacker M, Altman T, et al: **EcoCyc: a comprehensive database of *Escherichia coli* biology.** *Nucleic Acids Res* 2011, **39:**D583-590.

2. Cherry JM, Hong EL, Amundsen C, Balakrishnan R, Binkley G, Chan ET, Christie KR, Costanzo MC, Dwight SS, Engel SR, et al: ***Saccharomyces* Genome Database: the genomics resource of budding yeast.** *Nucleic Acids Res* 2012, **40:**D700-705.

3. Nookaew I, Jewett MC, Meechai A, Thammarongtham C, Laoteng K, Cheevadhanarak S, Nielsen J, Bhumiratana S: **The genome-scale metabolic model iIN800 of *Saccharomyces cerevisiae* and its validation: a scaffold to query lipid metabolism.** *BMC Syst Biol* 2008, **2:**71.

4. Herrgard MJ, Swainston N, Dobson P, Dunn WB, Arga KY, Arvas M, Bluthgen N, Borger S, Costenoble R, Heinemann M, et al: **A consensus yeast metabolic network reconstruction obtained from a community approach to systems biology.** *Nat Biotechnol* 2008, **26:**1155-1160.

5. Kanehisa M, Goto S, Sato Y, Furumichi M, Tanabe M: **KEGG for integration and interpretation of large-scale molecular data sets.** *Nucleic Acids Res* 2012, **40:**D109-114.

6. Schomburg I, Chang A, Placzek S, Sohngen C, Rother M, Lang M, Munaretto C, Ulas S, Stelzer M, Grote A, et al: **BRENDA in 2013: integrated reactions, kinetic data, enzyme function data, improved disease classification: new options and contents in BRENDA.** *Nucleic Acids Res* 2013, **41:**D764-772.

7. Wieczorke R, Krampe S, Weierstall T, Freidel K, Hollenberg CP, Boles E: **Concurrent knock-out of at least 20 transporter genes is required to block uptake of hexoses in *Saccharomyces cerevisiae*.** *FEBS Lett* 1999, **464:**123-128.

8. Förster J, Gombert AK, Nielsen J: **A functional genomics approach using metabolomics and *in silico* pathway analysis.** *Biotechnol Bioeng* 2002, **79:**703-712.

9. Gombert AK, Moreira dos Santos M, Christensen B, Nielsen J: **Network identification and flux quantification in the central metabolism of *Saccharomyces cerevisiae* under different conditions of glucose repression.** *J Bacteriol* 2001, **183:**1441-1451.

10. Verho R, Londesborough J, Penttila M, Richard P: **Engineering redox cofactor regeneration for improved pentose fermentation in *Saccharomyces cerevisiae.*** *Appl Environ Microbiol* 2003, **69:**5892-5897.

11. Saloheimo A, Rauta J, Stasyk OV, Sibirny AA, Penttila M, Ruohonen L: **Xylose transport studies with xylose-utilizing *Saccharomyces cerevisiae* strains expressing heterologous and homologous permeases.** *Appl Microbiol Biotechnol* 2007, **74:**1041-1052.

12. Karhumaa K, Garcia Sanchez R, Hahn-Hagerdal B, Gorwa-Grauslund MF: **Comparison of the xylose reductase-xylitol dehydrogenase and the xylose isomerase pathways for xylose fermentation by recombinant *Saccharomyces cerevisiae*.** *Microb Cell Fact* 2007, **6:**5.

13. Guijarro JM, Lagunas R: ***Saccharomyces cerevisiae* does not accumulate ethanol against a concentration gradient.** *J Bacteriol* 1984, **160:**874-878.

14. Takahashi S, Kuzuyama T, Watanabe H, Seto H: **A 1-deoxy-D-xylulose 5-phosphate reductoisomerase catalyzing the formation of 2-C-methyl-D-erythritol 4-phosphate in an alternative nonmevalonate pathway for terpenoid biosynthesis.** *Proc Natl Acad Sci U S A* 1998, **95:**9879-9884.

15. Seemann M, Bui BT, Wolff M, Tritsch D, Campos N, Boronat A, Marquet A, Rohmer M: **Isoprenoid biosynthesis through the methylerythritol phosphate pathway: the (E)-4-hydroxy-3-methylbut-2-enyl diphosphate synthase (GcpE) is a [4Fe-4S] protein.** *Angew Chem Int Ed Engl* 2002, **41:**4337-4339.

16. Rohdich F, Zepeck F, Adam P, Hecht S, Kaiser J, Laupitz R, Grawert T, Amslinger S, Eisenreich W, Bacher A, Arigoni D: **The deoxyxylulose phosphate pathway of isoprenoid biosynthesis: studies on the mechanisms of the reactions catalyzed by IspG and IspH protein.** *Proc Natl Acad Sci U S A* 2003, **100:**1586-1591.

17. Wolff M, Seemann M, Tse Sum Bui B, Frapart Y, Tritsch D, Estrabot AG, Rodrı́guez-Concepción M, Boronat A, Marquet A, Rohmer M: **Isoprenoid biosynthesis via the methylerythritol phosphate pathway: the (E)-4-hydroxy-3-methylbut-2-enyl diphosphate reductase (LytB/IspH) from *Escherichia coli* is a [4Fe–4S] protein.** *FEBS Letters* 2003, **541:**115-120.

18. Trinh CT, Unrean P, Srienc F: **Minimal *Escherichia coli* cell for the most efficient production of ethanol from hexoses and pentoses.** *Appl Environ Microbiol* 2008, **74:**3634-3643.

19. Carlson R, Srienc F: **Fundamental *Escherichia coli* biochemical pathways for biomass and energy production: identification of reactions.** *Biotechnol Bioeng* 2004, **85:**1-19.
